# Giving a Rat’s: Acquisition of Baseline Health Data on Native Australian Rodents During a Conservation Translocation

**DOI:** 10.3390/ani16193044

**Published:** 2026-09-27

**Authors:** Fiona Knox, Jill M. Austen, Claire R. Sharp, Timothy H. Hyndman, Jane P. Wesson, James K. Douch, Paola K. Vaz, Nahiid Stephens, Sarah Keatley, Amanda Ash, Colleen Sims, Saul Cowen, Kelly Rayner, Sean Garretson, Siobhon Egan, Kristin Warren, Lian Yeap, Rebecca Vaughan-Higgins

**Affiliations:** 1 School of Veterinary Medicine, Murdoch University, 90 South Street, Murdoch, WA 6150, Australia; knoxfiona@gmail.com (F.K.); jill.austen@dpird.wa.gov.au (J.M.A.); c.sharp@murdoch.edu.au (C.R.S.); t.hyndman@murdoch.edu.au (T.H.H.); j.wesson@murdoch.edu.au (J.P.W.); n.stephens@murdoch.edu.au (N.S.); s.keatley@murdoch.edu.au (S.K.); a.ash@murdoch.edu.au (A.A.); siobhon.egan@murdoch.edu.au (S.E.); l.yeap@murdoch.edu.au (L.Y.); 2 Asia-Pacific Centre for Animal Health, Melbourne Veterinary School, The University of Melbourne, Parkville, VIC 3010, Australia; james.douch@hdr.mq.edu.au (J.K.D.); pvaz@unimelb.edu.au (P.K.V.); 3 Biodiversity and Conservation Science, Western Australia Department of Biodiversity Conservation and Attractions, Wildlife Place, Woodvale, WA 6026, Australia; colleen.sims@dbca.wa.gov.au (C.S.); saul.cowen@dbca.wa.gov.au (S.C.); kelly.rayner@dbca.wa.gov.au (K.R.); sean.garretson@dbca.wa.gov.au (S.G.); 4 School of Biological Sciences, University of Western Australia, Crawley, WA 6009, Australia

**Keywords:** reintroduction, wildlife health, disease risk analysis, surveillance, pathology, parasitology, stress

## Abstract

Little is known about the baseline health status of Australia’s native rodents. The resultant knowledge gaps are problematic for wildlife translocations, as assessing disease risk is integral to translocation planning and outcomes. A disease risk analysis was conducted for the conservation translocation of native rodents to Dirk Hartog Island, Western Australia. This analysis resulted in a recommendation for a multidisciplinary project to reduce knowledge gaps about the health status of Shark Bay mice (*Pseudomys gouldii*) and greater-stick-nest rats (*Leporillus conditor*). Between 2021 and 2022, 130 Shark Bay mice and 122 greater stick-nest rats were translocated to Dirk Hartog Island. Mucosal swabs, whole blood, faeces, and ectoparasites were collected from a subset of these individuals. Molecular testing for four infectious agents was performed, parasite burdens and haematological parameters were examined, and relationships between these variables, health metrics and post-translocation short-term survival were assessed. Necropsies with routine histological assessment on translocation mortalities were performed where feasible. Several indicators of non-infectious factors contributing to morbidity or mortality were found, highlighting the need to iteratively evaluate translocation strategies. There was also an apparent absence of some host-specific parasites, suggesting that active approaches to parasite conservation during translocations should be considered. Molecular evidence of herpesviruses, a paramyxovirus, *Bartonella* sp., *Trypanosoma* sp. and *Mycoplasma* spp. were detected among the translocated animals. Although not associated with morbidity or short-term mortality in this study, the disease potential of these agents is still present. Overall, these results contribute important new data on health baselines for both species, which will assist in informing future conservation planning.

## Simple Summary

There is limited knowledge about the health of wild native Australian rodents. This complicates decision-making when trying to establish new populations of native rodents by moving individuals to new locations. This study of Shark Bay mice and greater stick-nest rats moved to Dirk Hartog Island in Western Australia examined various aspects of native rodent health. It examined whether the rodents had any evidence of infection by several disease agents, investigated parasites carried by these rodents and considered health outcomes during these human-assisted movements. While several disease agents were found, none were clearly linked to poor health. Meanwhile, health problems not caused by infectious disease agents were identified. Overall, the project provides essential data on the health of these rodent species.

## 1. Introduction

Australia’s native rodents form an ecologically significant and diverse component of the continent’s biodiversity. Despite undergoing a disproportionately high rate of extinction and declines in population sizes and ranges since European settlement, they maintain a low conservation profile [1,2,3]. The resulting knowledge deficiencies limit the ability to implement evidence-based decision-making when developing conservation strategies. With considerable gaps in the understanding of Australian native rodent ecology, knowledge of the parasites of these species, and the impact of these on host health, are equally lacking. This is particularly the case for rodents occupying semi-arid and arid environments, where assumptions of equivalence with their mesic and synanthropic counterparts are likely inappropriate [4,5]. Consequently, it is evident that baseline health data for Australian rodents is needed so that the health threats to rodents, sympatric species, and humans can be adequately factored into species conservation planning.

Establishing new populations or supplementing existing populations through translocations, is increasingly being utilised to enhance the conservation trajectory of several Australian rodent species [6,7]. This practice is inherently attached to a biosecurity risk. The life history traits of some rodent species increase their ability to acquire, maintain and transmit parasites in their populations, while the One Health significance of parasites harboured by some members of the order are internationally well-established [8,9,10,11,12]. Where translocations supplement existing rodent species networks, biosecurity risks could be amplified through increased spatial overlap of phylogenetically related species, which may increase the availability of naïve and susceptible hosts for a parasite to exploit [12,13]. Gold standard translocation planning necessitates robust consideration of these disease risks [14]. However, rodent life histories are diverse, and avoiding erroneous equivalence assumptions requires the availability of specific baseline health data, which is lacking for most Australian native rodents.

The Shark Bay mouse (SBM; *Pseudomys gouldii*) and the greater stick-nest rat (GSNR; *Leporillus conditor*) are two native Australian rodent species that have been translocated to several sites across Australia in response to population declines and extirpation of both species from the Australian mainland [15,16]. These species are currently listed as Vulnerable under Australia’s Environment Protection and Biodiversity Conservation Act 1999. Remnant populations on Bernier Island off the Western Australian coast (SBM) and on the East and West Franklin Islands off the coast of South Australia (GSNR) have, over the last few decades, supported translocations to establish new populations [15,16,17]. This includes recent translocations to Dirk Hartog Island (DHI) off the coast of Western Australia for an ecological restoration project known as “Return to 1616”. Four rodent species (SBM; GSNR; desert mouse, *Ps. desertor*; and heath mouse, *Ps. shortridgei*) were initially incorporated into translocation planning for “Return to 1616” [18], although *Ps. desertor* and *Ps. shortridgei* were subsequently removed from translocation plans. Although these species are being reintroduced to an area they once inhabited, they are also supplementing existing rodent species networks on DHI. Three rodent species (ash-grey mouse [AGM], *Ps. albocinereus*; sandy inland mouse [SIM], *Ps. hermannsburgensis*; and non-native house mouse [HM], *Mus musculus*) were found on the island prior to commencement of the project [18].

Recent analyses have highlighted the variable success of Australian rodent translocations. Predation, suboptimal habitat and climatic conditions at release sites, stress, and poor release protocols, have been implicated in translocation failures of both SBMs and GSNRs [16,17,19]. Diseases, particularly those with infectious aetiologies, have rarely been identified as contributing to translocation failure of either species, although respiratory pathologies of unknown aetiology correlated with high mortality rates in a prior GSNR translocation [16]. Their small size, rapid carcass decay under adverse climatic conditions, and susceptibility to scavenging/predation, frequently obviate the ability to perform gross necropsy and/or histopathological assessment of mortalities, which may obscure the role of health states in translocation outcomes. Despite captive breeding in several zoological collections, there are limited baseline health data available for either species to provide objective health measures or to determine parasites of concern for translocations.

Prior to translocating four rodent species to DHI, a disease risk analysis (DRA) was conducted. There were significant uncertainties identified during the DRA process. Limited baseline health data was available for the rodent species proposed for translocation, or for other species within the *Pseudomys* genus. Therefore, sampling at the time of translocation was recommended to address these knowledge gaps.

Previous studies of Australian rodents have identified several infectious agents with pathogenic potential, four of which were targeted for sampling. These included two viral families (*Orthoherpesviridae* and *Paramyxoviridae*), one protozoal genus (*Trypanosoma*), and one bacterial genus (*Mycoplasma*). Herpesviruses (family: *Orthoherpesviridae*) are DNA viruses that induce largely host-specific sub-clinical infections in immunocompetent natural hosts. However, immunosuppression or cross-species transmission may precipitate severe disease [20]. Rodent herpesviruses include murine betaherpesviruses (e.g., *Muromegalovirus* spp.) and murine gammaherpesviruses (e.g., *Rhadinovirus muridgamma4*), but numerous novel rodent herpesviruses have been discovered in recent decades, including one from a deceased sandy inland mouse from DHI [21,22]. Paramyxoviruses (PMVs) (family: *Paramyxoviridae*) are single-stranded RNA viruses and include Mossman virus (*Narmovirus mossmanense*) and J-virus (*Jeilongvirus queenslandense*), both first discovered in rodents in Queensland [23,24,25]. Natural and experimental infections of these paramyxoviruses in rodents have been associated with interstitial pneumonia and severe, diffuse haemorrhagic lung lesions [23,24,25]. While PMVs have not previously been detected in Western Australian rodents, their disease potential warrants epidemiological investigation. *Trypanosoma* spp. have been previously identified in the SBM and other native rodents and *T. lewisi* has been implicated in extinctions of native rodents on Christmas Island [26,27,28]. While some *Trypanosoma* spp. are associated with severe clinical disease and are of biosecurity significance for the international movement of animals (e.g., *T. evansi* in livestock), the impact on host-health of Australian trypanosomes is uncertain [28,29,30]. Finally, *Mycoplasma* spp., particularly *M. pulmonis*, have long been associated with respiratory and other diseases in rodents [31]. Wild HM have previously tested seropositive to *M. pulmonis* in Australia, while a *Mycoplasma*-associated disease outbreak in a captive native rodent colony has been recorded [32,33]. Infection with *Mycoplasma* spp. is often subclinical but stress (inherent during translocations of wild animals) may induce clinical disease, with the potential to influence translocation outcomes [31].

The aim of this study was to obtain baseline health information on SBM and GSNR populations subject to translocation to DHI, to inform future conservation planning of these species. Our objectives were to characterise physical and haematological parameters, characterise parasite occurrence and burdens, screen for the presence of four infectious agents (herpesviruses, paramoxyviruses, *Trypanosoma* spp., and *Mycoplasma* spp.) and ascertain causes of short-term mortality across the translocated animals. We hypothesised that translocation source groups would differ in measured variables and that the presence of infectious agents would negatively impact health and/or short-term survival post-translocation.

## 2. Materials and Methods

### 2.1. Translocation: Animal Capture and Physical Examination

Comprehensive details of translocation strategies for both species have been published [34,35]. Briefly, SBMs were live trapped using baited Elliott traps over a period of three nights in April 2021 and four nights during April–May 2022 at Northwest Island and Bernier Island, respectively. Greater stick-nest rats were live trapped using a mix of methods: baited Elliott traps, baited Sheffield cage traps, or hand-caught over a period of four nights in May 2021 and two nights in May 2022 from Salutation Island or the East and West Franklin Islands, respectively (Figure 1). Rodents were placed in individual clean cloth bags to assess their suitability for translocation. Suitability for translocation was determined on sex, age, reproductive condition, body condition score (BCS), body weight, and health examination, including ectoparasite burdens, presence of cataracts and other detectable physical abnormalities. Animals selected for translocation were weighed and microchipped, their morphometrics were recorded, and they were placed in transport boxes containing food and insulating substrate for transportation.

### 2.2. Sample Collection Under Anaesthesia

Following arrival on DHI, all rodents received a visual welfare check to assess for mortality, injuries or overt signs of distress. In accordance with the translocation proposal, 13–16 animals of each species per translocation were removed from transport boxes for radio-collar application under general anaesthesia (GA). Only adults not detectably pregnant or lactating, meeting minimum weight criteria (percentage of radio-collar weight to bodyweight; collar ≤ 3% bodyweight for SBM, ≤3.5% bodyweight for GSNR), and desired sex ratios, were collared. These individuals were subjected to comprehensive health examinations and targeted sample collection under GA. Individuals with abnormalities identified on the visual welfare check were also examined under GA, sampled where possible, and treatment administered where required.

Anaesthesia was induced and maintained using isoflurane (ISO, Veterinary Companies of Australia, Artarmon, NSW, Australia) in 100% medical grade oxygen delivered via face mask or nose cone by a portable inhalational anaesthetic machine (‘Stinger’, Advanced Anaesthetic Specialists, Australia). Following induction, single or combined swabs for infectious agent testing were collected by swabbing the oral, conjunctival, urogenital and rectal spaces using sterile flocked swabs (Copan, Brescia, Italy; Table 1). Swabs were then placed into sterile 2 mL cryovials either dry, in saline or in viral transport medium (VTM; PathWest, Claremont, WA, Australia) for infectious agent polymerase chain reactions (PCR; Table 1). Whole blood was collected for haematology and molecular testing from the lateral coccygeal vein with a 25–29 G cut-off butterfly needle, with a heat pack placed over the region for 1–2 min prior, to facilitate blood flow. Physical examination was performed by a veterinarian. Any detected abnormalities were documented. Complete sample collection was not performed, or was discontinued, if the animal was considered unstable under GA or if there were animal welfare concerns. Following full anaesthetic recovery, animals were returned to translocation boxes, released and radio-tracked daily where possible until collar removal or a mortality event (up to 28 days for SBM and up to 39 days for GSNR; [34,35]).

### 2.3. Haematology

Packed cell volume (PCV) was measured following centrifugation of microhaematocrit tubes at 7500× *g* for a total of five minutes at room temperature (ZipCombo, LW Scientific, Lawrenceville, GA, USA), and total solids (TS) was measured with a refractometer (Sper Scientific, Scottsdale, AZ, USA). Fixed blood smears were stained with a May–Grünwald–Giemsa stain and examined using light microscopy for white blood cell (WBC) differential counts (100 or 200 cells were counted depending on smear quality and size), cell morphology and haemoparasites at both 400× and 1000× magnification. Total estimated WBC counts (cells/μL) were estimated by calculating the average number of WBCs across 10 monolayer fields at 400× magnification and multiplying this value by 1600 [36,37]. Neutrophil to lymphocyte (N:L) ratios were also determined.

### 2.4. Parasitology

Faeces were collected opportunistically from translocated animals from transport boxes or handling bags into vials containing 10% neutral-buffered formalin (NBF; Rowe Scientific, Wangara, WA, Australia). These samples were assessed using zinc sulphate flotation (specific gravity 1.2) and malachite green staining of smears for helminth ova and protozoal oocysts. Identification was by light microscopy according to standard morphological criteria, including size, shape, wall morphology and internal structures, and classified to the family or genus level where possible.

Where ectoparasites were visualised, representative samples were collected and stored in 70% ethanol. For animals under GA, a consistent observer subjectively assessed ectoparasite burdens as none, low, moderate or heavy. Ectoparasites were examined using light or stereo microscopy. For fleas, specimens were identified to the genus or species level where possible using keys provided by Dunnet and Nardon [38]. Ticks were identified using keys provided by Roberts [39] and Roberts [40]. Where mites were collected, these were sent to the Commonwealth Scientific and Industrial Research Organisation (CSIRO), Black Mountain, for identification and prepared for examination as described in Halliday [41]. The exception was for mites from the Franklin Islands that were identified to the family level using keys provided in Domrow [42]. Lice were identified to the family level using the keys of Kim and Ludwig [43].

### 2.5. Molecular and Sequence Analysis for Selected Infectious Agents

Nucleic acids from whole blood stored in EDTA were extracted according to manufacturer instructions using a MasterPure Complete DNA and RNA Purification Kit (Lucigen, Middleton, WI, USA; GSNR) or from Whatman FTA Elute cards (GE Healthcare, Chicago, IL, USA; SBM). PCR-grade water was added to extend sample volumes to 200 μL where necessary. A nested PCR protocol was used to detect a 959 bp region of the *Trypanosoma* spp. 18S rRNA gene [29,44] (Table 2). Additionally, nucleic acids extracted from splenic tissue of two SBM and one GSNR were tested using this same protocol. Reactions were carried out in a total volume of 25 μL, consisting of 12.5 μL of Promega GoTaq Green Mastermix (Promega, Madison, WI, USA), 0.4 μM of each of forward and reverse primers (external primers S-LF and S-762R, and internal primers S-825F and S-LIR; Table 2), 8.5 μL (primary) and 9.5 μL (secondary) of water, and 2 μL (primary) and 1 μL (secondary) of template. Cycling conditions were as follows: the primary assay was 94 °C for 5 min, 50 °C for 2 min, and 72 °C for 4 min, 40 cycles of (94 °C for 30 s, 52 °C for 30 s, and 72 °C for 1 min), and 72 °C for 7 min; the secondary assay was 94 °C for 5 min, 50 °C for 2 min, and 72 °C for 4 min, 40 cycles of (94 °C for 40 s, 55 °C for 30 s, 72 °C for 1 min), and 72 °C for 7 min.

A *Bartonella* PCR was performed on a single extracted DNA sample from SBM blood with consistent intraerythrocytic organisms on blood smear examination. The protocol targeted 370–450 bp of the 16S rRNA–23S internal transcribed spacer (ITS) gene regions [45] (Table 2). Reactions were carried out in a total volume of 25 μL each containing 1× buffer (KAPA Biosystems, Wilmington, MA, USA), 2.0 mM MgCl_2_, 0.4 μM of each primer 438s and 1100as, 0.25 mM of each dNTP, 0.5 U of Taq (KAPA Biosystems) and 2 μL of template. Cycling conditions were as follows: 95 °C for 3 min, 40 cycles of (95 °C for 15 s, 66 °C for 15 s, and 72 °C for 18 s), and 72 °C for 5 min.

Two swabs (a combined conjunctival–oral swab and a urogenital–rectal swab) from each rodent were tested using a nested pan-*Orthoherpesviridae* PCR that targets a conserved region of the herpesvirus DNA polymerase catalytic subunit (DPOL) [46]. A QIAamp Viral RNA mini kit (QIAGEN, Clayton, VIC, Australia) was used to extract nucleic acids from these swabs according to manufacturer instructions. Three primers are used in the primary round (DFA-F, ILK-F and KG1-R) and two primers in the secondary round (TGV-F, IYG-R). Reactions were carried out in a total volume of 25 μL consisting of 5 μL of 5x buffer (Promega, Madison, WI, USA), 1 mM of MgCl_2_, 0.2 mM of each dNTP, 0.4 μM (primary) or 0.8 μM (secondary) of each primer, 1 U GoTaq Flexi DNA polymerase (Promega) and 5 μL of template. Cycling conditions were as follows: a primary round of 95 °C for 5 min, 45 cycles of (95 °C for 30 s, 46 °C for 30 s, and 72 °C for 90 s), and 72 °C for 2 min; a secondary round of 95 °C for 5 min, 45 cycles of (95 °C for 30 s, 46 °C for 30 s, and 72 °C for 60 s), and 72 °C for 2 min.

To detect *Mycoplasma* spp. and paramyxoviruses, nucleic acids were extracted from a single combined conjunctival, oral, urogenital and rectal swab using a PureLink Viral RNA/DNA Mini kit (Invitrogen, Carlsbad, CA, USA) according to manufacturer instructions.

For paramyxoviruses, semi-nested RT-PCR protocols targeting a 584 bp region of the *Paramyxoviridae* L. gene (PAR RT-PCR) and a 494 bp region of the *Respirovirus*, *Morbillivirus* and *Henipavirus* L. gene (RMH RT-PCR) were used as described by Tong et al. [47], with minor modifications including reduced reaction volumes and use of commercial master mixes. For both the PAR-PCR and RMH-PCR, the Superscript III One-Step RT-PCR System with Platinum Taq (Invitrogen) was used in the primary PCR.

For the PAR RT-PCR, reactions were carried out in a total volume of 20 μL, consisting of 0.8 μL of RTTaq, 1 μM of each primer PAR-F1 and PAR-R, 2 μL of template, 10 μL of mastermix, and 3.2 μL water. For the secondary PCR, reactions were carried out in a total volume of 20 μL consisting of 1 μM of each primer (PAR-F2 and PAR-R), 2 μL of template (from the first round reaction), 10 μL of Platinum Green PCR 2x Mastermix (Invitrogen, Carlsbad, CA, USA) and 4 μL of water. Cycling conditions were as follows: a primary round of 60 °C for 1 min, 45 °C for 30 min, 94 °C for 2 min, 40 cycles of (94 °C for 15 s, 50 °C for 30 s, and 72 °C for 30 s), and 72 °C for 7 min; a secondary round of 94 °C for 2 min, 40 cycles of (94 °C for 15 s, 50 °C for 30 s, and 72 °C for 30 s), and 72 °C for 7 min.

For the RMH RT-PCR, primary reactions were carried out in a total volume of 20 μL consisting of 0.8 μL of RTTaq, 1 μM of each primer RMH-F1 and RMH-R, 2 μL of template, 10 μL of mastermix and 3.2 μL of water. For the secondary round, reactions were carried out in a total volume of 20 μL consisting of 1 μM of each primer (RMH-F2 and RMH-R), 2 μL of template (from the first round reaction), 10 μL of Platinum Green PCR 2x Mastermix and 4 μL of water. Cycling conditions were identical to those of the primary and secondary rounds of the PAR assays.

For *Mycoplasma* spp., a PCR targeting a conserved 270 bp region of the Mollicutes 16S rRNA gene was utilised [48]. Reactions were carried out in a total volume of 20 μL consisting of 1 μM of each primer (GPO-3 and MGSO), 1 μL of template, 10 μL of Platinum Green PCR 2x Mastermix and 5 μL of water. Cycling conditions were as follows: 94 °C for 2 min, 10 cycles of (94 °C for 60 s, 65 °C for 60 s [decreasing by 1 °C every cycle], and 72 °C for 60 s), and then 30 cycles of (94 °C for 60 s, 55 °C for 60 s, and 72 °C for 60 s).

All PCR primers are shown in Table 2. All extractions included negative controls and PCRs included positive and negative controls. PCR products were separated by agarose gel electrophoresis using standard techniques. Amplicons close to the predicted size were excised from the gel, purified, added to forward and reverse primers, and then submitted to the Australian Genome Research Facility (AGRF) for Sanger sequencing.

All nucleotide sequences were compared with sequences in GenBank (National Center for Biotechnology Information), using the nucleotide basic alignment search tool (BLASTn), and assessed for similarity with other sequences in the database for closest hits. Translated BLAST (BLASTx) was additionally used for these comparisons for the herpesvirus DPOL sequence. For phylogenetic analysis, sequences obtained through BLASTn and manual searches of GenBank were aligned using multiple sequence comparison by log-expectation (MUSCLE) or ClustalW (for *Bartonella*) in MEGA12 [49] with default settings and trimmed. The best models for phylogenetic analysis were determined through the model function in MEGA12, selecting the model with the lowest Bayesian Information Criterion values. The appropriate model was then used to create each phylogenetic tree, with confidence in relationships evaluated by a bootstrap analysis with 1000–10,000 replicates.

**Table 2:** PCR assays, target genes and primers used in this study to test samples from translocated rodents for infectious agents.

Assay	Target Gene	Primers	Primer Sequences (5′–3′)	Amplicon Size (bp)
Pan-*Orthoherpesviridae* PCR [46]	DNA polymerase catalytic subunit UL30	DFA ILK KG1 TGV IYG	GAYTTYGCNAGYYTNTAYCC TCCTGGACAAGCAGCARNYSGCNMTNAA GTCTTGCTCACCAGNTCNACNCCYTT TGTAACTCGGTGTAYGGNTTYACNGGNGT CACAGAGTCCGTRTCNCCRTADAT	~200
Mollicutes (pan-*Mycoplasma* PCR) [48]	16S rRNA	GPO-3 MGSO	GGGAGCAAACAGGATTAGATACCCT TGCACCATCTGTCACTCTGTTAACCTC	270
Pan-*Paramyxoviridae* RT-PCR [47]	*Paramyxoviridae* L. gene	PAR-F1 PAR-F2 PAR-R	GAAGGITATTGTCAIAARNTNTGGAC GTTGCTTCAATGGTTCARGGNGAYAA GCTGAAGTTACIGGITCICCDATRTTNC	584
*Respirovirus*, *Morbillivirus* and *Henipavirus* genera RT-PCR [47]	*Respirovirus*, *Morbillivirus* and *Henipavirus* L. gene	RMH-F1 RMH-F2 RMH-R	TCITTCTTTAGAACITTYGGNCAYCC GCCATATTTTGTGGAATAATHATHAAYGG CTCATTTTGTAIGTCATYTTNGCRAA	494
*Trypanosoma* spp. PCR [29,44]	18S rRNA	S-762R S-LF S-825F S-LIR	GACTTTTGCTTCCTCTAATG GCTTGTTTCAAGGACTTAGC ACCGTTTCGGCTTTTGTTGG ACATTGTAGTGCGCGTGTC	959
*Bartonella* spp. PCR [45]	16S rRNA–23S ITS	438s 1100as	GGTTTTCCGGTTTATCCCGGAGGGC GAACCGACGACCCCCTGCTTGCAAAGC	370–450

### 2.6. Necropsy and Histopathology

All detected mortalities during or post-translocation were subject to detailed necropsy by a veterinarian or ecologist (carcass state permitting) and gross findings were documented. Representative samples of tissue were divided; one segment was frozen, and the other was placed into 10% NBF. For histopathological analyses, fixed tissue was trimmed and processed in ethanol and xylene before paraffin embedding. Five micrometre sections were stained with haematoxylin and eosin. Ancillary staining was used where indicated. Examination of slides was performed by a board-certified veterinary pathologist (NS).

Immunohistochemistry examination for *Toxoplasma gondii* was performed as follows. Following deparaffinisation and rehydration, sections were heat-treated in a microwave oven with Tris-EDTA buffer for antigen recovery. Sections were then treated with 3% hydrogen peroxide for 10 min and with normal goat serum for 10 min to block nonspecific binding. This was followed by a 30 min incubation in *To. gondii* rabbit polyclonal antibody (ThermoFisher Scientific, Waltham, MA, USA) and a 30 min incubation in Dual Link secondary antibody (Dako, Carpinteria, CA, USA). Slides were developed with 3,3′-diaminobenzidine (DAB+) substrate-chromogen (Dako, Carpinteria, CA, USA) for 3 min followed by staining with haematoxylin and then blueing solution.

### 2.7. Statistical Analyses

Statistical analyses were performed in R version 4.4.0 [50] using the epiR, tidyverse, Hmisc and outliers packages.

Haematology parameters for both rodent species were checked for outliers using the Dixon Q test. For all variables and species, descriptive statistics and histograms were generated (Figures S1 and S2).

The apparent prevalence of parasites and infectious agents was calculated with 95% confidence intervals using the Wilson score interval, assuming perfect test sensitivity and specificity. Tests of association were carried out with a statistical significance set at α = 0.05, using Pearson’s Chi-squared tests with Yates’ continuity correction (where all expected frequencies were ≥5) or Fisher’s exact test (where any expected frequency was <5) for categorical variables. For continuous variables, normality was assessed using the Shapiro–Wilk normality test. Univariate analyses (Welch two-sample *t*-test or Wilcoxon rank sum test) were then used to test associations. Correlations between continuous variables (N:L ratios, percentage weight change, PCV, and TS) were measured with Pearson’s r in GSNRs.

## 3. Results

### 3.1. Sample Population

One hundred and thirty SBMs and 122 GSNRs were translocated to DHI during 2021 and 2022. Of these, 30 SBMs and 29 GSNRs were anaesthetised for either radio-collar application or welfare assessment. These 59 animals underwent more comprehensive veterinary assessment and targeted sample collection (Figure 2; Table 3).

### 3.2. Physical Examination

Of the 59 animals assessed, three SBMs required GA for welfare assessments, all due to likely capture-related acute tail degloving. No welfare assessments under GA were required for GSNRs. One SBM anaesthetic in 2021 was abandoned as a stable anaesthetic plane could not be reached. These abnormalities resolved on recovery and this individual was released, but subsequently tested positive for a *Mycoplasma* sp.

Poor BCS (BCS ≤ 2/5) was the most common physical abnormality of GSNRs and was significantly more common in GSNRs from Salutation Island (Fisher’s exact *p* = 0.034; Table 3), with all anaesthetised GSNRs from the Franklin Islands-sourced group in good body condition (BCS > 2/5). Acute weight loss was noted across both source groups of GSNRs but was significantly greater in the Franklin Islands-sourced group (W = 0, *p* < 0.001). Precautionary subcutaneous fluids were administered to all anaesthetised GSNRs in 2022 as weight loss was thought to be at least partly attributable to dehydration. Although mean body weight differed between sites for SBMs, there was marked dimorphism between the two island locations, and BCS did not significantly differ between sites.

Other physical examination findings included a GSNR that presented with perioral dermatitis accompanied by alopecia and crusting, and another GSNR that presented with multiple small ~1 mm raised nodules on the preputial sheath, without visible evidence of accompanying inflammation. In both instances, sampling was not permissible under animal ethics and permit arrangements. One SBM presented with a complete unilateral cataract with periocular dermatitis and blepharospasm, but the contralateral eye was considered normal.

### 3.3. Haematology

Haematology results from anaesthetised rodents are reported in Table 4. Small sample sizes from SBMs prevented meaningful analyses, while statistical testing indicated several haematological parameters were significantly associated with demographic or clinical parameters in GSNRs (Figure 3a–d). For GSNRs, PCVs were higher in Franklin Islands-sourced individuals than Salutation Island-sourced individuals (t[df = 18.66] = 3.58, *p* = 0.002; Figure 3a), while TS and N:L ratios were significantly higher in males than females (TS: t[df = 17.31] = −2.32, *p* = 0.03; N:L ratios: t[df = 19.88] = −2.36, *p* = 0.03; Figure 3b,c). Total estimated WBCs were higher in individuals in poor body condition than those in good body condition (t[df = 4.78] = −2.74, *p* = 0.04). Correlations between PCV and percentage weight change (*r*(22) = −0.72 [*p* < 0.001]) and N:L ratios (*r*(22), = 0.49, *p* = 0.02) were identified, with PCV increasing as animals lost more weight and as N:L ratios increased (Figure 3d). Similarly, N:L ratios also correlated with percentage weight change (*r*(22) = −0.51, *p* = 0.015) (Figure 3d), with N:L ratios increasing as animals lost more weight.

Intraerythrocytic bacilliform inclusions consistent with the appearance of a *Bartonella* sp. were identified on microscopic examination of one SBM blood sample from 2022. Subsequent molecular testing detected *Bartonella* DNA in whole blood, with the amplified sequence of the 16S–23S ITS aligning with other species from the *B. queenslandensis* clade, but with relatively low sequence identity (89.6%) across the cognate region of other members of this clade (GenBank accession number PV344459; Figure 4). There was no indication of clinical compromise nor haematological perturbations in this individual, and it survived the post-release period until collar removal. *Laelaps lybacia* mites were the only ectoparasite collected from this individual. No evidence of other haemotropic microorganisms were detected through blood smear examination of any other individual.

### 3.4. Parasitology

One hundred and thirty-two faecal samples (89 SBMs and 43 GSNRs) were examined during 2021 and 2022, with gastrointestinal parasites identified in 30.3% (*n* = 40/132) of samples, being 27% of SBM samples (*n* = 24/89; 95% CI 18.84–37) and 37.2% of GSNR samples (*n* = 16/43; 95% CI 24.38–52.14). Across both species, protozoa were the most prevalent parasites and were the only gastrointestinal parasite taxon detected in GSNR samples (Table S1). *Trichomonidae* flagellates/cysts (likely to be *Tritrichomonas*) dominated the faecal parasitology of SBMs while *Entamoeba* and unidentified protozoan oocysts dominated in GSNRs. Although the latter displayed few morphological features for identification, they were not considered to be consistent with known pathogens *Giardia* or *Cryptosporidium* spp. Helminth ova were found only in SBM samples. Cestode ova were found in a single SBM sample from Bernier Island (morphology consistent with a *Hymenolepis* sp.) and spiruroid ova were found in two SBM samples from Northwest Island (morphology of one consistent with *Protospirura* or *Mastophurus*). There were no detectable associations of faecal parasites with demographic or health parameters in either rodent species.

The number of different ectoparasite species collected from the natural Bernier Island SBM population was greater than the translocated Northwest Island population. The mite *L. lybacia* was the only ectoparasite recovered from the Northwest Island-sourced group. Mites (*L. lybacia* and *Mesolaelaps australiensis*), fleas (*Xenopsylla* sp.), and ticks (*Haemaphysalis humerosa*) were recovered from the Bernier Island-sourced group. Lice were not recovered from any SBMs.

Lice, mites and fleas were collected from GSNRs across both source groups. *Xenopsylla* sp. were recovered across both source locations, while *Echidnophaga myrmecobii* and *Parapsyllus australiacus* specimens were collected only from Franklin Islands-sourced GSNRs. *Pa. australiacus*, usually associated with seabirds [38], was found on a single individual and is likely a spurious record, possibly due to GSNRs utilising shearwater burrows on the island. *Laelaps rothschildi* was identified from a Salutation Island-sourced GSNR in 2021, while in 2022, mites were only identified to the family level as *Laelapidae* mites. *Hoplopleuridae* lice, a likely species being *Hoplopleura leporilludis* [53], were found across both source groups. Mixed infections of all three ectoparasite groups were common.

### 3.5. Testing for Selected Infectious Agents

*Orthoherpesivirdae*, *Paramyxoviridae*, *Trypanosoma* spp. and *Mycoplasma* spp. nucleic acids were all detected in at least one rodent species during these translocations. However, there were no significant associations between these results and sex, location, BCS, or short-term survival (Table 5). Only one animal (a GSNR) tested positive for more than one infectious agent (testing positive for both herpesvirus and *Mycoplasma* by PCR).

*Trypanosoma* sequences were detected in four GSNRs from the Franklin Islands, with no detectable clinical abnormalities in these individuals who all survived the immediate post-release period (Figure 5). The 18S rRNA gene sequences obtained were 100% identical across their readable length (734–786 nucleotides; GenBank accession number PZ298589), and clustered with sequences found in non-Australian murids. The closest identities were to a *Trypanosoma* sp. detected in *Mus mahomet* from Ethiopia (MZ703261; 98.6% identity; Table 6; [54]). This latter sequence was placed by the authors in the *Herpetosoma* subgenus, but peripheral to the *T. lewisi* subclade [54]. All *Trypanosoma*-positive individuals had detectable flea (*n* = 4/4), mite (*n* = 2/4) or louse (*n* = 2/4) infestations, but no ticks were observed. Ectoparasites recorded from *Trypanosoma*-positive individuals were *Hoplopleuridae* lice (likely *H. leporilludis*), *Laelapidae* mites and *E. myrmecobii*. Ectoparasites were not tested for trypanosomes in this study.

Two novel herpesvirus sequences were found at low (3.8%; *n* = 1/26) and moderate (20.7%; *n* = 6/29) prevalences in SBMs and GSNRs, respectively. The readable section of the SBM sequence was too short (54 nucleotides) to precisely delineate phylogenetic relationships, but the closest hits were to murid betaherpesvirus sequences (Table 6). A second herpesvirus was detected in GSNRs from both source locations (Table S2). The GSNR herpesvirus sequence shares closest alignments to a herpesvirus from a brown rat (*Rattus norvegicus*) from China (KX987256: 85% nucleotide identity; 167/196 nucleotides; 91% amino acid identity for the corresponding protein sequence; Table 6). The top 30 closest hits for this sequence in GenBank through BLASTn were murid rhadinoviruses (*Gammaherpesvirinae*). Although one herpesvirus-positive SBM and GSNR died post-release, neither carcass was suitable for necropsy. Half (*n* = 3/6) of the herpesvirus-positive GSNRs were in poor body condition and 83.3% (*n* = 5/6) of herpesvirus-positive rodents were from Salutation Island. Body condition score and location differences in prevalence were not statistically significant (*p* = 0.08 and *p* = 0.18, respectively). All herpesvirus-positive GSNRs were positive on oral–conjunctival swabs, with two also positive on urogenital–rectal swabs. The SBM herpesvirus-positive animal was only positive on a urogenital–rectal swab.

A likely novel paramyxovirus sequence (530 nucleotides, GenBank Accession number PV856277), detected by the PAR RT-PCR, was retrieved from a Franklin Islands-sourced GSNR, sharing closest (79.5%) identity to a rodent *Jeilongvirus* (Meliandou uranomys virus isolate AFGN2, GenBank nucleotide accession: OK623366; Table 6). Although the positive GSNR was known to have died post-release, there was no evidence of paramyxovirus-consistent pathology on histopathology.

*Mycoplasma* sequences were detected from both species and all locations (Table 6). Phylogenetic analysis placed the sequences within the *Mycoplasma* genus, but internal relationships within the genus had low bootstrap support and hence are not reported. Sequences from three SBMs were 100% identical and shared closest alignments with *M. neurolyticum* (AB680669; 96.82% identity; Table 6). Sequences obtained from GSNRs included two identical sequences from different source groups, with closest alignments with an uncultured bat *Mycoplasma* from Central America (MH245146; 95.91% identity; Table 6), a sequence closely aligned to *M. spermatophilum* (MF769617; 99.44% identity; Table 6) and another sequence with closest but distant alignments to another uncultured bat *Mycoplasma* from Central America (MK353862; 78.01% identity; Table 6). Two *Mycoplasma*-positive individuals died; one during harvest (SBM) and one post-translocation (GSNR). The SBM had no histological evidence of pathology associated with *Mycoplasma* infections in murids. The GSNR carcass was not suitable for necropsy and was the only individual that was test-positive for more than one infectious agent in this study (it was also PCR positive for a herpesvirus). Clinical abnormalities were restricted to one *Mycoplasma*-positive SBM that demonstrated respiratory abnormalities under GA that resolved on recovery. The post-release survival time of this individual is unknown.

### 3.6. Mortalities

Five and four post-translocation mortalities were detected during the radio-tracking period across both years in SBMs and GSNRs, respectively. However, only two GSNR carcasses in 2021, and one GSNR carcass in 2022, were in a suitable state for necropsy. Advanced decomposition (*n* = 1) or lack of an intact carcass (*n* = 5) prevented necropsy of all other carcasses. All post-translocation mortalities were associated with evidence of predation and/or scavenging by reptiles (varanids and snakes) or birds of prey [34,35].

The cause of death for all recovered GSNR carcasses subject to necropsy and histopathology was consistent with predation. One deceased individual in 2021 had evidence of a mature cortical cataract. Although the examined GSNR from 2022 was PMV-positive by PCR, histopathology did not reveal any significant abnormalities to suggest the paramyxovirus contributed to morbidity or predation of this individual.

In addition to post-translocation mortalities, two SBMs that died during handling for translocation on Bernier Island in 2022 were also examined. Necropsy and histopathology revealed subcutaneous haemorrhage associated with the microchip site in both individuals despite appropriate placement; this was mild in one individual and extensive in the other. Both individuals demonstrated diffuse pallor of musculature, and passive hyperaemia or hypostasis in several organs. Unilateral sub-capsular adrenal haemorrhage was found in one individual. Martius Scarlet Blue-positive histochemical staining of cardiomyocytes suggestive of per-acute cardiomyocyte degeneration, without evidence of contraction band formation (via phosphotungstic acid haematoxylin histochemistry) was found in both individuals. Protozoal cysts, up to 100 μm in diameter and containing crescent-shaped bradyzoites, were identified in the skeletal muscle of one SBM with minimal associated inflammatory infiltrate. Immunohistochemical staining for *Toxoplasma* was negative. Although further testing was not performed, based on the cyst size, a *Sarcocystis* sp. was considered the most likely.

## 4. Discussion

This study provides a multidisciplinary baseline health assessment of SBM and GSNR populations translocated to DHI, contributing essential data to inform future conservation planning of these species. The study evaluated physical and haematological parameters, characterised parasite occurrence and burdens, then examined associations of these parameters with health and short-term mortality in translocated animals. Consistent with our initial hypotheses, differences in physical parameters, haematological values, and parasite occurrence were found between translocation source groups. The findings also suggest the need for continued efforts to minimise stress in translocation operations. The molecular evidence of *Trypanosoma* sp., *Bartonella* sp., herpesviruses, *Mycoplasma* spp., and a paramyxovirus detected in this study all represent novel host records. Although not clearly associated with impacts on health or short-term mortality, some of these infectious agent groups have previously been associated with reduced fitness in rodent populations, or with realised or predicted zoonotic potential [24,28,56].

Few physical abnormalities were found in SBMs translocated to DHI, with none considered significant enough to prevent release of animals. Poor body condition and acute weight loss were the major findings in GSNRs sourced from Salutation Island and the Franklin Islands, respectively. Combined with haematology findings, the physical findings in GSNRs provided several indications of stress in this species, despite mitigation efforts, particularly in the Franklin Islands-sourced group. Physiological stress is an expected consequence of wildlife translocations and has previously been implicated in rodent translocation failures [16,57]. A relative neutrophilia in GSNRs was found, and correlations of the N:L ratio with acute weight loss suggest catecholamine-driven neutrophil redistribution. Elevated PCVs also correlated with acute weight loss, suggesting splenic contraction and/or fluid losses may have led to a relative erythrocytosis. The cause of the acute weight loss, particularly pronounced in the Franklin Islands-sourced group, is unknown, but was presumed to be due to fluid deficits. This may have occurred through a combination of anorexia (despite provision of ‘wet food items’ during translocation) and excessive micturition during transport, the latter previously noted as a stress response in GSNRs [Perth Zoo, unpublished data; ref. [58]]. The differences observed between translocation source groups may have been influenced by the longer transport times experienced by the Franklin Islands-sourced group relative to animals from Salutation Island. Although clinical signs of dehydration were absent and mean TS was within the range of prior research, subcutaneous fluids were administered to a subset of Franklin Islands-sourced individuals prior to release as a precaution. These haematological perturbations merit further exploration, as both stress and fluid deficits could significantly impair physiological responses post-release. In the interim, due to probable translocation-induced perturbations, the reported haematological results for both species warrant cautious interpretation, and for this reason, the calculation of reference ranges was excluded from this study.

Parasite data was also obtained, with several parasites identified as likely to be of conservation significance. For example, *L. lybacia*, which had only been described from one female specimen prior to this translocation, may be threatened alongside their SBM hosts, but there is no empirical data to support this [41]. Parasites with complex life cycles might be at particular risk of silent extinction where translocation is a critical component of recovery plans. Supporting this, all parasites with complex life cycles in this study were found in remnant populations. Others, such as gastrointestinal protists, may be normal flora, disruption of which could have unpredictable effects on host immunity and health [59,60,61]. Although some parasites are at similar, if not greater, risk of co-extinction with their hosts, this is rarely acknowledged in translocation and ex situ conservation efforts, and some authors have noted the need for a shift towards integrating active parasite conservation approaches into translocation planning frameworks, including DRAs [62,63,64]. For example, there was no evidence of the species-specific flea *Acanthopsylla franklinensis* being present in GSNRs (most significantly, its absence from the remnant Franklin Islands-sourced group), suggesting conservation of this parasite might be warranted in future. However, determining which parasites to control or conserve requires baseline data collection and synthesis, particularly as many rodent-associated parasites are known to transmit diseases of One Health concern [62,65].

In conducting health assessments for the translocated animals, several previously unreported host–parasite associations were identified, demonstrating that SBM and GSNR carry a diverse range of infectious agents that are present in other murids globally. The first record of *Trypanosoma* (*Herpetosoma*) in the blood of four GSNRs was expected, as molecular studies have demonstrated the ubiquity of the rodent-associated *Herpetosoma* subgenus, including among Australian rodents [26,27,54,66]. All positive records were from the Franklin Islands. This may be due to small sample sizes but could also suggest transmission was restricted to this location, perhaps dictated by the availability of suitable vectors (which are currently unknown). This study did not identify evidence of infection in SBMs despite prior detections in this species [26]. This was likely due to small sample sizes. The *Trypanosoma* genus includes species responsible for important production-limiting diseases of livestock [67], fatal or disabling diseases of humans [68,69], and population declines of wildlife [30], with many species representing biosecurity risks during the transboundary movement of animals [67]. The type species of the *Herpetosoma* subgenus, *T. lewisi*, is a rare zoonosis that has been implicated in the extinction of two native Australian rodents [28,67,70]. Experimental evidence indicates the capacity of some members of this subgenus to induce anaemia and reproductive failure, and severe disease exacerbated by immunosuppression in rodents, while documented disease in humans, although rare, suggests pathogenic and cross-species transmission potential for some *Herpetosoma* spp. [70,71,72]. However, no evidence of morbidity associated with the detection of *Trypanosoma* sequences was found in this study. Life histories of the *Trypanosoma* genus are diverse. Many *Herpetosoma* are currently considered host-specific and of minimal pathogenic significance in natural hosts, though recent research has prioritised cataloguing *Herpetosoma* diversity over ascertaining their health impacts [54,73]. Although current evidence indicates the translocation of *Herpetosoma* spp. with rodent hosts in Australia is likely to be of low consequence, there is still an insufficient understanding of the health effects or potential host-range of *Herpetosoma* in Australian rodents.

The *Bartonella* sequence from the SBM is likely a novel species within the *B. queenslandensis* clade, which has previously been identified from *Melomys* spp. and several introduced and native *Rattus* spp. in eastern Australia [74,75]. This novel species is the first identification of this *Bartonella* clade from wildlife in Western Australia. A study of feral cats on DHI reported the absence of any *Bartonella* infection, but sympatric rodents were not examined [76]. The lack of clinical compromise in this individual is consistent with an infection of a natural reservoir host [77,78]. Although population level studies using molecular techniques would be required to elucidate both the natural host range and disease associations, disease has not yet been associated with the detection of *Bartonella* spp. in Australian wildlife [79]. There is currently no evidence to suggest that the *B. queenslandensis* clade is zoonotic, and rodent-associated *Bartonella* infections of humans are currently considered rare [80]. Typically transmitted by haematophagous arthropods, the vector in this case is unknown. Members of the clade have been identified in fleas and mites (including *Laelaps* sp.) from rodent hosts in other parts of the world [81,82,83]. Mites from the haematophagous *Laelaps* genus were carried by the SBM, but several potentially competent ectoparasite vectors were also present in the source population. Although only one individual in this study demonstrated haemoparasites on blood smear examination, cytological detection of haemoparasites has a low sensitivity in chronic infections, and without application of broader molecular testing, this study likely underreports haemoparasite presence in both populations [67,84].

The first detections of herpesviruses in SBMs and GSNRs in this study reflects the ubiquity and diversity of vertebrate herpesviruses, and research biases that have historically neglected small mammals [21,85,86,87]. The low nucleotide (85%) and amino acid (91%) identity of the GSNR herpesvirus sequence with the closest sequences in GenBank suggest that it may be a novel herpesvirus species that falls within the *Gammaherpesvirinae* subfamily. As this sequence has never been detected in other host species, and as gammaherpesviruses are typically host-specific, the GSNR may be a natural host of this herpesvirus. Although the closest hits of this virus were other murine rhadinoviruses, further analysis of a larger portion of the genome would be required to clearly delineate taxonomic relationships, while the position of the SBM sequence within the *Orthoherpesviridae* family remains uncertain. Following initial productive infection, herpesviruses typically establish persistent, latent infections in immunocompetent natural hosts, but disease may be precipitated by immunosuppression [88,89]. Lymphoproliferative disorders [88,90,91] and reproductive failure [92,93] have been associated with murine gammaherpesvirus infections, while reduced longevity has been associated with gammaherpesviruses in wild rodent hosts [56]. It remains to be seen if the detected sequence represents a virus with similar pathogenic potential.

Chronic stress can trigger reactivation of latent herpesviral infections, and this may account for five of six herpesvirus-positive GSNRs originating from Salutation Island [20]. The translocation from Salutation Island corresponded with a population decline of the GSNR from high densities, and relatively elevated faecal glucocorticoids, a marker of stress, were recorded in the population at the time of translocation [94]. It was assumed that the Salutation GSNR population had exceeded its carrying capacity prior to translocation, and the poor condition of the animals from this location supports this. There is no evidence to suggest that the GSNR herpesvirus contributed to these population dynamics, as cyclical irruptive population dynamics is considered typical of this population. Density-dependent transmission may also account for these findings. Further longitudinal studies may be able to clarify these relationships. Nevertheless, the results highlight the need for cautious decision-making when translocating populations experiencing demographic fluctuations. Despite the absence of overt disease, external stressors may trigger enhanced shedding of infectious agents, which may increase biosecurity risks [95].

No evidence of Sendai virus (*Respirovirus muris*) or *M. pulmonis*, both important respiratory pathogens of laboratory rodents, was found in this study. This is consistent with the results of studies screening for antibodies to these agents in wild HMs in Australia [33,96,97]. Several genetic sequences consistent with other *Mycoplasma* spp. were identified, some sharing closest sequence identity to *M. neurolyticum*, the causative agent of ‘rolling disease’ in experimental infections of laboratory rodents [98]. Larger gene segment sequencing is required to clarify these relationships. Non-haemotropic *Mycoplasma* infections of laboratory and pet rodents, frequently as opportunistic infections, may induce respiratory disease [31,99], polyarthritis [100], and reduced fertility or reproductive failure [31,101]. Cases of overt disease in wild rodents are rarely documented, although subclinical pathology has been identified [102]. While one *Mycoplasma*-positive individual demonstrated respiratory abnormalities during anaesthesia in this study, these respiratory complications could not be further characterised, and *Mycoplasma* infection cannot be conclusively linked to these findings.

Similarly, the novel PMV detected cannot be linked to adverse health findings. It should be noted that the brain of the affected GSNR was not available for histopathology, and so examination for changes consistent with a viral agent with neurotropism was not possible. No histopathology consistent with a viral agent with lymphotropism, endotheliotropism, respirotropism, or hepatotropism was seen in the affected GSNR. Nevertheless, the finding is noteworthy, as PMVs are among the few viral families that have been identified as potentially pathogenic to wild rodent hosts in Australia to date. For example, J-virus was first isolated from moribund HMs in North Queensland that demonstrated haemorrhagic lung lesions [24]. Several PMVs are of known or emerging significance to human and animal health, with some rodent PMVs considered to have high zoonotic spill-over potential [103,104,105]. Accordingly, the pathogenic potential of this PMV should not be overlooked. This is particularly relevant given the novel sympatric overlap of closely related species due to translocation and the known capacity of PMVs to cross species-barriers [106]. It is possible that this study underestimated the prevalence of PMVs in this population given the potentially low sensitivity of the broadly reactive semi-nested RT-PCRs used, compared to more specific PMV RT-PCR. Nonetheless, use of such broadly reactive RT-PCR assays is routine for the detection of novel PMVs [47,104]. Further research into the diversity and epidemiology of rodent PMVs in Australia is needed to better understand this finding and to inform future translocations.

It is important to acknowledge that the limitations of this study likely restricted the ability to discern health effects associated with these infectious agents. All individuals sampled were considered healthy for translocation, and radio-collared individuals subjected to targeted sampling were generally the heaviest of their source groups. These selection biases possibly excluded animals suffering clinical disease, thereby distorting health and survival outcomes. Additionally, a balance was required between obtaining adequate sample numbers and minimising additional handling time and stress associated with sample collection. The small sample sizes and lack of longitudinal data, combined with the complex disease dynamics of the selected infectious agents, mean that the potential population health significance of these agents should not be overlooked. Predilection sites of these infectious agents are also unknown, so our sampling may have missed infected individuals. Further, four GSNRs from Salutation Island produced amplicons of the expected size with the pan-*Paramyxoviridae* RT-PCR, but sequences could not be resolved and, without validation, were treated as negative results. Therefore, our results may underrepresent agent diversity. Future research should aim to further characterise these agents (e.g., viral isolation and broader genetic analysis) and their effects.

Noting limitations, the lack of association of infectious agents with morbidity or short-term mortality does not negate their disease potential. Carcass retrieval was difficult, and although all post-translocation mortalities in this study were attributed to likely predation, scavenging could not be excluded [34,35]. Where necropsy was possible, a mature cataract, that presumably reduced visual acuity, could have contributed to the predation susceptibility of at least one GSNR. Cataracts have previously been identified as being of population health concern to GSNRs, with a high prevalence noted in both captive and wild populations in the 1990s [[107], Perth Zoo, unpublished data]. Although aetiologies were not determined, genetics and/or environmental factors were postulated [[108], Perth Zoo, unpublished data]. Despite implementing cataract screening during translocation, mydriatics which would have enhanced detection of cataracts were not utilised, and should be considered in future studies.

Unfortunately, aetiologies for the sudden deaths of two SBMs that occurred during handling could not be determined. Stress-induced cardiomyopathy, adrenal crisis or haemorrhagic shock could not be confirmed. Adrenal haemorrhage may lead to acute adrenal insufficiency and is commonly associated with stress [109,110]. Typically, such stress-related haemorrhage presents as bilateral and florid, but in the SBMs, adrenal haemorrhage was unilateral and moderate. Other causes of sudden cardiac death (e.g., catecholamine-driven arrhythmias) also remain possible [111,112]. Since translocation, a further sudden handling death of an SBM without overt cause and no definitive histopathological findings has occurred on DHI. All three deaths reportedly occurred immediately after microchipping, but there was no evidence of incorrect microchip placement, and all handlers were experienced with this species. These findings underscore the potential risks of interventions involving capture and handling of this species and the need to balance monitoring and translocation objectives with these risks.

## 5. Conclusions

This study provides important baseline health data for GSNRs and SBMs, identifying several novel host–parasite associations. It reports the first known records of *Trypanosoma* sp., *Mycoplasma* spp., herpesviruses, *Paramyxoviridae* and *Bartonella* sp. in these species, and identifies several non-infectious factors that potentially contributed to morbidity or mortality. The detected infectious agents did not affect short-term translocation success, and imposition of screening to exclude infected individuals during future translocations is not supported by current evidence. However, their pathogenic potential remains unclear due to study limitations, the complex interplay of factors in disease dynamics, and the frequently subtle impacts of disease in wildlife populations. Ongoing monitoring, particularly continued investigation of future mortalities, will be important to evaluate the role these infectious agents have on wildlife health and the long-term success of translocations. This is particularly crucial considering the existing rodent networks on DHI and the significance of spatial and genetic proximity for enhancing the potential for parasite spillover. Although novel, the findings of this study are also reflective of the limited effort historically directed towards understanding the disease status of Australian native rodents and highlight the significant opportunities to expand and refine the current knowledge base. Recovery teams should consider supporting research to address these gaps, focusing this research on established populations. Greater stick-nest rats, SBMs and several other native rodent species are restricted to limited populations that are desired by multiple agencies to support translocation efforts. With effective coordination, planning and funding, implementation of research to build the health knowledge required to inform translocation strategies is feasible and would significantly expand the limited understanding of native rodent health in Australia.

## Figures and Tables

**Figure 1:**
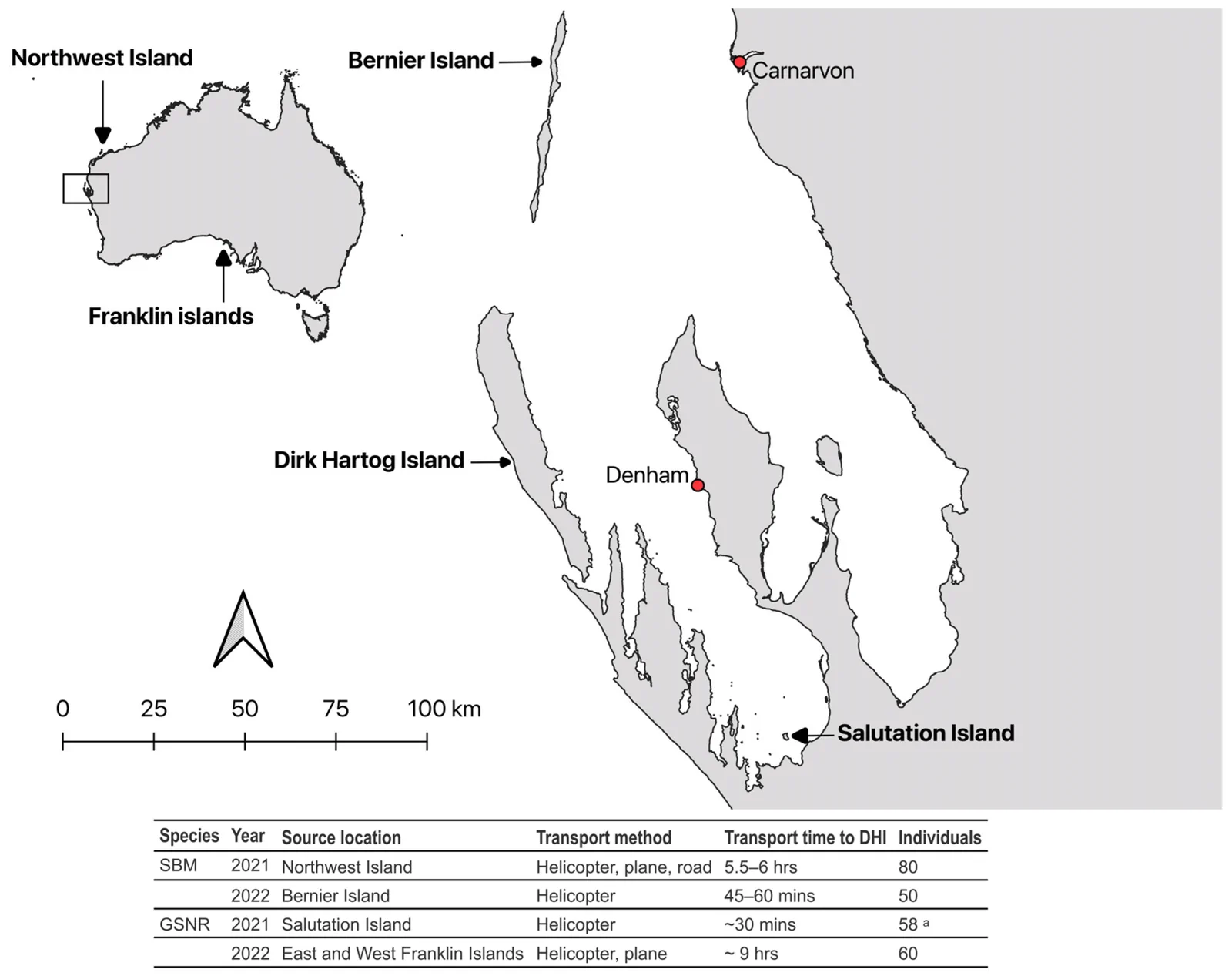
Source populations, transport methods, duration and number of individuals (Shark Bay mice [SBM] and greater stick-nest rats [GSNR]) translocated to Dirk Hartog Island (DHI) from 2021–2022. The two source populations for SBM were Bernier Island, a natural population of this species located in the Shark Bay region of Western Australia, and Northwest Island, located in the Montebello Island group. The Northwest Island population is a translocated population initially founded in 1999 from Bernier Island individuals (via captive breeding at Perth Zoo). For GSNR, individuals were sourced from the East and West Franklin Islands, providing natural populations of this species located off the coast of South Australia, and Salutation Island. Salutation Island is home to a translocated population initially founded in 1990 from animals sourced from the East and West Franklin Islands (via captive breeding at Monarto Zoo). A total of 130 SBMs and 122 GSNRs were translocated to DHI from these four source locations, with transport durations ranging from 30 min to approximately nine hours. ^a^ An additional four GSNRs were translocated from Salutation Island, indirectly, after being held in captivity near Denham for a radio-collar trial.

**Figure 2:**
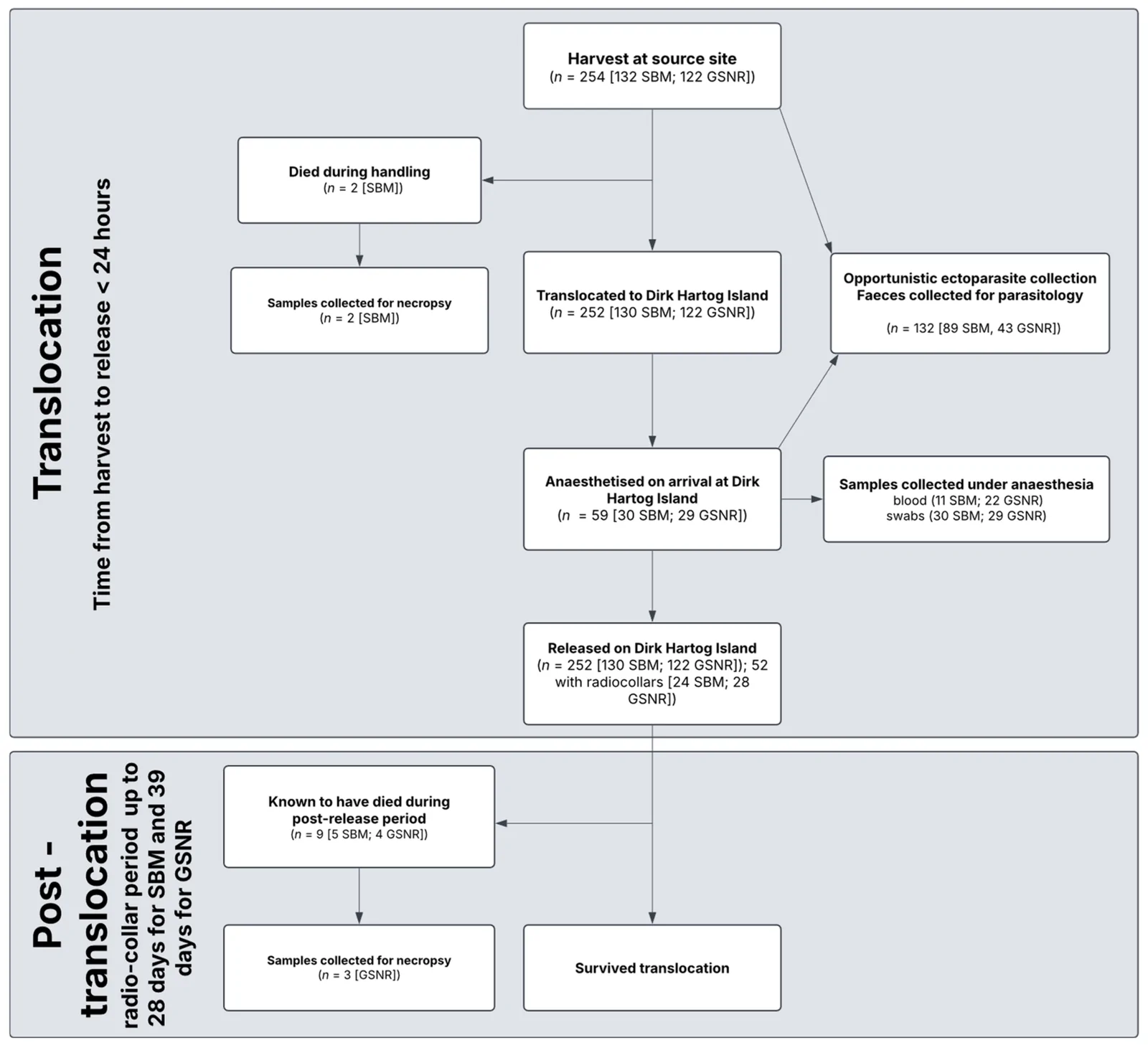
Timeline of sampling events conducted during and post-translocation.

**Figure 3:**
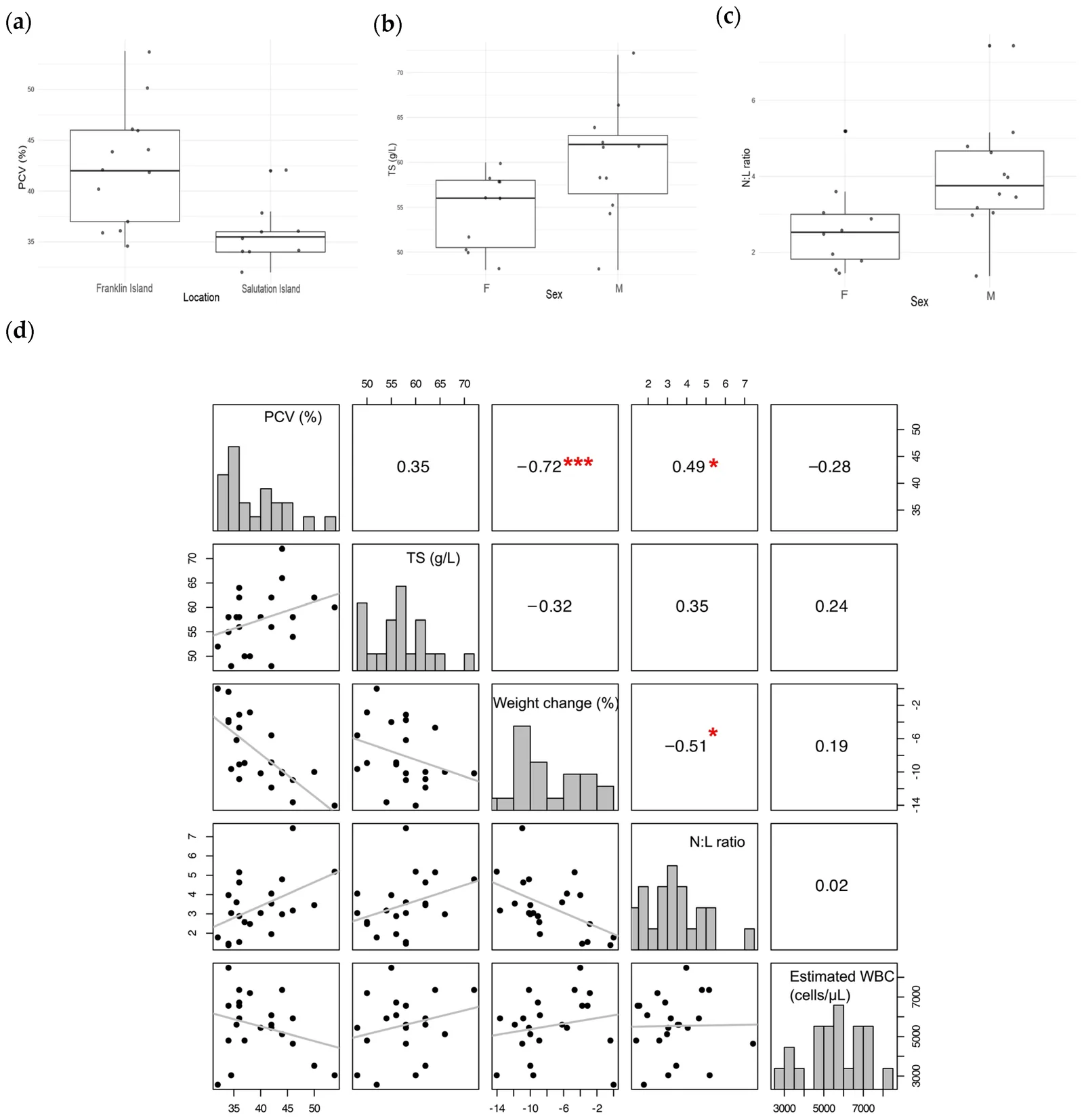
Haematological parameters where significance testing indicated differences or correlations with analysed variables for GSNRs: All boxplots show the median and interquartile range (IQR), with whiskers extending to 1.5× IQR. Raw data is overlaid: (**a**) boxplot of PCV by location, with measured PCV significantly higher in the Franklin Islands-sourced group than the Salutation Island-sourced group (*p* = 0.002); (**b**) boxplot of total solids by sex, with TS significantly higher in males than females (*p* = 0.03); (**c**) boxplot of N:L ratio by sex, with N:L ratios significantly higher in males than females (*p* = 0.03); and (**d**) correlogram of continuous parameters in GSNRs (PCV; TS g/L; weight change % and N:L Ratio). Pearson’s r values are in the upper right, with asterisks denoting significance values (*** *p* < 0.001; * *p* < 0.05). Histograms of each continuous variable are on the diagonal, while scattergrams and lines of best fit between continuous variables are in the lower-left quadrants. Strong and significant correlation was observed between PCV and weight change (%), while moderate and significant correlation was observed between N:L ratios and weight change (%) as well as PCV (%).

**Figure 4:**
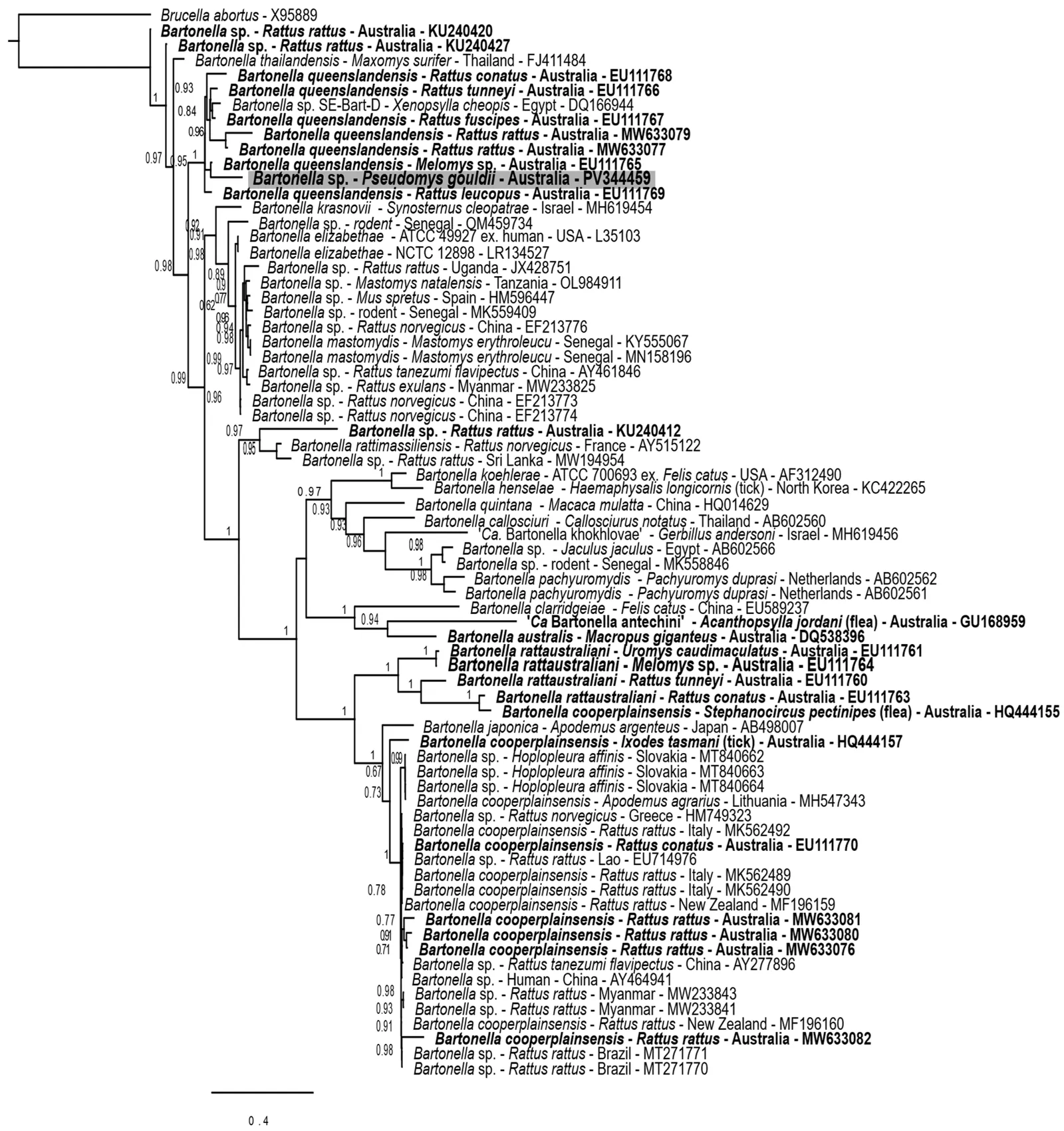
Phylogenetic tree of *Bartonella* nucleotide sequences identified in an SBM based on a segment of the 16S–ITS region. The sequence obtained from the present study (PV344459) is highlighted in the shaded box, while other sequences detected from Australian wildlife are in bold. The phylogenetic tree was developed in MEGA12. The phylogeny was inferred using the maximum likelihood with the HKY + F substitution model with 10,000 replicates. Node values, corresponding with bootstrap support, are shown for nodes with greater than 60% support and the scale bar indicates the number of substitutions per site.

**Figure 5:**
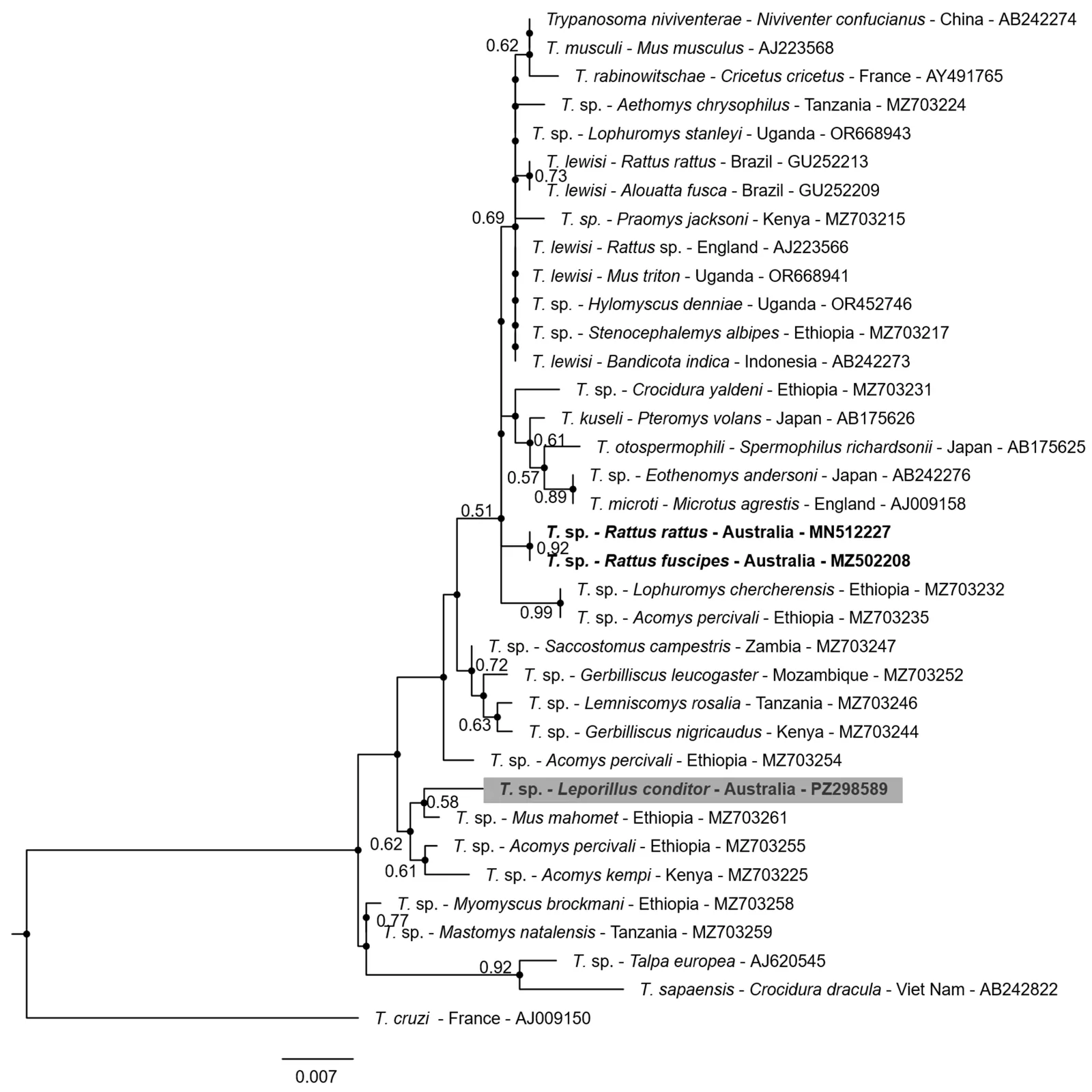
Phylogenetic tree of *Trypanosoma* sp. nucleotide sequences identified in GSNRs from the Franklin Islands based on a segment of the 18S rRNA gene (highlighted; GenBank Accession number PZ298589). Other sequences detected from rodents in Australia are in bold. The phylogenetic tree was developed in MEGA12 [49]. The phylogeny was inferred using the maximum likelihood method and Kimura 2 parameter model [55] with gamma distribution and invariant sites. The tree with the highest log likelihood is shown. Bootstrap support values (1000 replicates) are shown for nodes with greater than 50% support and the scale bar indicates the number of substitutions per site.

**Table 1:** Samples collected from rodents under general anaesthesia (GA) for either radio-collar application or welfare assessment during translocation to DHI. Four swabs in different storage media were collected from all animals where possible. These were utilised in targeted polymerase chain reactions (PCRs) for *Orthoherpesviridae* (herpesvirus), *Paramyxoviridae* (paramyxovirus) and *Mycoplasma* spp., and for archival purposes. Whole blood samples were collected for both haematology and *Trypanosoma* spp. testing. The small size of SBM limited blood volumes that could be safely acquired, so whole blood in ethylenediaminetetraacetic acid (EDTA) was collected only from GSNRs. PCV = packed cell volume; TS = total solids.

Sample	Media	Purpose	Storage Conditions
Ectoparasites	70% ethanol	Identification	Room temperature
Oral swab	Dry	Long-term storage	−20 °C in the field then −80 °C for long term storage
Combined oral, conjunctival, urogenital, rectal swab	0.9% Saline	Paramyxovirus and *Mycoplasma* PCRs	−20 °C in the field then −80 °C until analysis
Conjunctival–oral swab	VTM	Herpesvirus PCR	4 °C in the field then −80 °C until analysis
Urogenital–rectal swab	VTM	Herpesvirus PCR	As for conjunctival–oral swab
Blood	Heparinised microhematocrit tube Thin blood smear Whatman FTA Elute cards (GE Healthcare, Chicago, IL, USA) EDTA tube (GSNR)	PCV/TS measurement Cytology *Trypanosoma* PCR (SBM); archiving (GSNR) *Trypanosoma* PCR	Analysed on collection Methanol fixation; then, room temperature until May–Grünwald–Giemsa staining and analysis Room temperature in the field; then, −80 °C until analysis −20 °C in the field; then, −80 °C until analysis

**Table 3:** Results of physical examination of 59 rodents under general anaesthesia (GA) during translocation to DHI. BCS = body condition score; SD = standard deviation.

	Shark Bay Mouse		Greater Stick-Nest Rat	
Year (number examined under GA)	2021 (15)	2022 (15)	2021 (16)	2022 (13)
GA purpose (*n*)				
Radio-collar application (collars applied)	13 (12)	14 (12)	16 (15)	13 (13)
Welfare	2	1	0	0
Sex (*n*)				
Male	7	7	4	8
Female	8	8	12	5
BCS (*n*) [51]				
Poor (BCS ≤ 2/5)	1	0	6	0
Good (BCS > 2/5)	14	15	10	13
Mean body weight (g) (SD)	59.1 (7.2)	42.3 (6.6)	261 (18.6)	298.8 (19.9)
Male	63 (8.2)	48.4 (3.2)	250 (12.2)	307.5 (11)
Female	55.68 (4.2)	36.9 (2.6)	264.7 (19.3)	285 (24.2)
Mean weight change between capture and arrival (%) (SD)	−1.81 (2.77)	0.35 (3.23)	−4.06 (2.27)	−10.63 (1.66)
Male	−0.62 (1.64)	0.27 (3)	−3.67 (2.29)	−10.96 (1.26)
Female	−2.85 (3.21)	0.42 (3.63)	−4.19 (2.35)	−10.11 (2.22)
Dermal abnormalities (*n*)				
Faecal or urine matting/staining	1	1	-	-
Degloved tail	2	1	-	-
Old tail amputation	-	1	-	-
Circumferential bruise at tail base	-	1	-	-
Perioral dermatitis with alopecia and crusting	-	-	1	-
Alopecia without signs of dermatitis	-	-	1	1
Recent traumatic wounds	-	-	-	2
Preputial nodules (unknown aetiology)	-	-	-	1
Ectoparasite burden (*n*)				
Heavy	0	2	0	2
Moderate	1	4	6	4
Mild	4	7	8	5
None	10	2	2	2
Ocular abnormalities (*n*)				
Unilateral cataract with periocular dermatitis and blepharospasm	1	-	-	-
Oral abnormalities (*n*)				
Dental abnormalities	1	-	-	-
Genital abnormalities (*n*)				
Abnormal vaginal discharge	-	1	-	1
Respiratory abnormalities (*n*)				
Increased respiratory tract sounds	1	2	-	-
Cardiac abnormalities (*n*)				
Sinus arrhythmia	-	-	-	1

**Table 4:** Haematology parameters from rodents under GA during translocation to DHI. Blood collection was not attempted in SBMs in 2021, resulting in small sample sizes. In SBMs, blood collection was unsuccessful from three individuals and could not be performed for welfare reasons from one individual. Additionally, a processing failure meant PCV/TS was not recorded for one individual. In GSNRs, blood collection was unsuccessful from five individuals and not attempted on two individuals due to GA length, while a processing failure meant TS was not measured for one individual. No published reference ranges were available for SBMs. Histograms of these results can be found in Figures S1 and S2. CI = confidence interval.

	SBM						GSNR						GSNR Reference Ranges
Measure	*n*	$\bar{x}$	SD	Med.	Min.	Max.	*n*	$\bar{x}$	SD	Med.	Min.	Max.	(A) Tulk et al. 2016 (90% CI) [52] (B) Species 360 ^a^
PCV %	10	47.25	2.85	47.25	42.5	52	22	39.67 **	5.79	37.5	32	53.8	(A) 32–36 (B) 25.1–41
TS (g/L)	10	58.6	4.33	59	52	66	21	57.48	6.08	58	48	72	(A) (Total protein) 51.62–61.29 (B) (Total protein) 23–79
Total estimated WBCs (10^9^/L)	10 *	3.41	0.95	3.28	2.08	5.12 *	22	5.53 **	1.54	5.6	2.56	8.48	(A) 9.65–12.85 (B) 1–15
Lymphocyte %	11	85.78	4.59	85	77.5	91.5	22	24.34 **	7.65	23.25	11.5	39.5	(A) 26.66–37.33 (B) 17.2–85.8
Neutrophil %	11	10.86	4.42	10	5	18.5	22	72 **	9.15	73	54.5	85.5	(A) 58.18–69.60 (B) 9.2–78.6
Monocyte %	11	1.27	0.75	1.5	0	2.5	22	2.34 **	1.48	2	0	5	(A) 3.02–5.96 (B) 1–10.5
Eosinophil %	11	1.64	0.98	2	0	3.5	22	1.05 **	1.1	0.75	0	3.5	A) 1.39–2.78 B) 0–6
Basophil %	11	0.18	0.34	0	0	1	22	0	0	0	0	0	-
Band %	11	0.23	0.41	0	0	1	22	0.14	0.27	0	0	1	-
Annular %	11	0.05	0.15	0	0	0.5	22	0.14	0.35	0	0	1.50	-
N:L Ratio	11	0.13	0.06	0.12	0.05	0.24	22	3.37	1.47	3.11	1.38	7.43	(A) 2.79–4.62 (B) -

* one outlier excluded. ** sample mean was outside a reference range reported in prior research. ^a^ Species 360 data were downloaded on 7 September 2024.

**Table 5:** Prevalence of selected infectious agents among individuals (SBMs and GSNRs) that were released on DHI. Relevant clinical abnormalities in test-positive individuals are noted, along with results for tests of association with sex, location, BCS and short-term survival. + = positive; HV = herpesvirus; Tryp. = *Trypanosoma* sp.; Myco = *Mycoplasma* spp.; PMV = paramyxovirus; AP = apparent prevalence. *p* = Fisher’s exact *p*-value.

Variable	HV +	HV AP (95% CI)	Tryp. +	Tryp. AP (95% CI)	Myco. +	Myco. AP (95% CI)	PMV +	PMV AP (95% CI)
**GSNR**								
Total positive	6/29	20.7 (9.8–38.4)	4/23 ^a^	17.4 (7–37.1)	4/29	13.8 (5.5–30.6)	1/29 ^b^	3.4 (0.2–17.2)
Male	3/12	*p* = 0.67	1/12	*p* = 0.32	2/12	*p* = 1	1/12	*p* = 0.41
Female	3/17		3/11		2/17		0/17	
Salutation Island	5/16	*p* = 0.18	0/10 ^a^	*p* = 0.1	2/16	*p* = 1	0/16 ^b^	*p* = 0.45
Franklin Islands	1/13		4/13		2/13		1/13	
Morbidity or mortality in test-positive individuals	Peri-oral dermatitis (*n* = 1) Poor BCS (*n* = 3) Post-release mortality (*n* = 1) Sinus arrhythmia (*n* = 1)		None		Poor BCS (*n* = 1) Post-release mortality (*n* = 1) Sinus arrhythmia (*n* = 1)		Post-release mortality (*n* = 1)	
Known to have died	*n* = 1	*p* = 1	*n* = 0	*p* = 1	*n* = 1	*p* = 0.47	*n* = 1	*p* = 0.14
Known to have survived	*n* = 4	*p* = 0.61	*n* = 4	*p* = 0.54	*n* = 3	*p* = 1	*n* = 0	*p* = 0.24
BCS	>2 = 3/23 ≤2 = 3/6	*p* = 0.08	>2 = 4/19 ≤2 = 0/4	*p* = 1	>2 = 3/23 ≤2 = 1/6	*p* = 1	>2 = 1/23 ≤2 = 0/6	*p* = 1
**SBM ^c,d^**								
Total positive	1/26	3.8 (0.2–18.9)	0/11	0 (0–25.9)	2/30	6.7 (1.8–21.3)	0/30	0 (0–11.4)
Male Female	1/11	*p* = 0.43	0/5	-	2/14	*p* = 0.21	0/14	-
	0/15		0/6		0/16		0/16	
Northwest Island	0/11	*p* = 1	-	-	2/15	*p* = 0.48	0/15	-
Bernier Island	1/15		0/11		0/15		0/15	
Morbidity or mortality in test-positive individuals	Post-release mortality (*n* = 1)		-		Degloved tail (*n* = 1) Unstable anaesthetic (*n* = 1)		-	
Known to have died	*n* = 1	*p* = 0.15	-	-	*n* = 0	*p* = 1	-	-
Known to have survived	*n* = 0	*p* = 0.38	-	-	*n* = 1	*p* = 1	-	-

^a^ One reported blood sample was collected on death of a GSNR for *Trypanosoma* PCR. The spleen of this individual was also tested and was negative by PCR. ^b^ Four GSNRs from Salutation Island produced bands of the expected size with the pan-*Paramyxoviridae* PCR, but sequencing was unable to confirm these individuals as true-positives. ^c^ As all bar one SBM had a good BCS, statistical testing for BCS associations was not performed for this species. ^d^ In addition to reported numbers, two SBMs that died during handling on Bernier Island were tested for all four infectious agents. One of these individuals tested positive for *Mycoplasma* by PCR (data not included above).

**Table 6:** Alignment table for molecular sequencing results obtained in this study. Nucleotide alignments were assessed for similarity with previously reported sequences in GenBank with closest matches reported.

Species	Location	Closest Match	Percent Identity (Query Cover %)
*Bartonella* 16S rRNA–23S ITS			
SBM	Bernier Island	*Bartonella* sp. SE-Bart_D (DQ166944)	92% (100%)
*Orthoherpesviridae* DNA polymerase catalytic subunit UL30 ^a^			
SBM	Bernier Island	*Muromegalovirus muridbeta1*—*Mus musculus*—USA—OP429142.	94.44% (100%)
GSNR ^b^	Salutation Island and Franklin Islands	Rat herpesvirus—*Rattus norvegicus*—China KX987256	85.2% (100%)
Mollicutes 16S rRNA ^c^			
SBM ^d^	Northwest Island and Bernier Island	*Mycoplasma neurolyticum*—AB680669	96.82% (100%)
GSNR ^e^	Salutation Island and Franklin Islands	Uncultured *Mycoplasma* sp.—*Rhynchonycteris naso*—Belize—MH245146	95.91% (100%)
GSNR	Salutation Island	Uncultured *Mycoplasma* sp.—Hairy-legged myotis (*Myotis keaysi*)—Belize. MK353862	78.01% (94%)
GSNR	Franklin Islands	*Mycoplasma spermatophilum*—human—China—MF769617	99.44% (100%)
*Paramyxoviridae* L. gene			
GSNR	Franklin Islands	Meliandou uranomys paramyxovirus isolate AFGN2—Guinea. OK623366	79.5% (97%)
*Trypanosoma* 18S rRNA			
GSNR ^f^	Franklin Islands	*Trypanosoma* sp.—*Mus Mahomet*—Ethiopia—MZ703261	98.6% (100%)

^a^ Full sequences are available in Table S2. ^b^ All sequences from GSNR across both locations were 100% identical. ^c^ Full sequences are available in Table S3. ^d^ All sequences retrieved from SBMs from both locations were 100% identical. ^e^ Two GSNR nucleotide sequences were 100% identical, one each from the Franklin Islands and Salutation Island. ^f^ All sequences from GSNRs were 100% identical.

## Data Availability

The data presented in this study are available on request from the corresponding author.

## Supplementary Materials

The following supporting information can be downloaded at https://www.mdpi.com/article/10.3390/ani16193044/s1: Figure S1: Histograms of haematology results of Shark Bay mice; Figure S2: Histograms of haematology results of greater stick-nest rats; Table S1: Faecal parasitology apparent prevalence (AP) across species and cohorts; Table S2: Nucleotide sequences obtained from amplification of the *Orthoherpesviridae* DNA polymerase catalytic subunit gene region from rodent swabs collected during this study; Table S3: Nucleotide sequences obtained from amplification of the Mollicutes 16S rRNA gene region from rodent swabs collected during this study.

## Abbreviations

The following abbreviations are used in this manuscript:

AGM	Ash grey mouse
AGRF	Australian Genome Research Facility
AP	Apparent prevalence
BCS	Body condition score
CSIRO	Commonwealth Scientific and Industrial Research Organisation
DHI	Dirk Hartog Island
DNA	Deoxyribonucleic acid
DPOL	DNA polymerase
DRA	Disease risk analysis
EDTA	Ethylenediaminetetraacetic acid
GA	General anaesthetic
GSNR	Greater stick-nest rat
HM	House mouse
IQR	Interquartile range
ITS	Internal transcribed spacer
NBF	Neutral buffered formalin
N:L	Neutrophil to lymphocyte
PCR	Polymerase chain reaction
PCV	Packed cell volume
PMV	Paramyxovirus
RMH	Respirovirus, Morbillivirus and Henipavirus
rRNA	Ribosomal ribonucleic acid
RNA	Ribonucleic acid
RT-PCR	Reverse transcription polymerase chain reaction
SBM	Shark Bay mouse
SIM	Sandy inland mouse
TS	Total solids
VTM	Viral transport media
WBC	White blood cell

## References

[1] Fleming P.A., Bateman P.W. (2016). The good, the bad, and the ugly: Which Australian terrestrial mammal species attract most research?. Mammal Rev..

[2] Roycroft E., MacDonald A.J., Moritz C., Moussalli A., Portela Miguez R., Rowe K.C. (2021). Museum genomics reveals the rapid decline and extinction of Australian rodents since European settlement. Proc. Natl. Acad. Sci. USA.

[3] Smith A.P., Quin D.G. (1996). Patterns and causes of extinction and decline in Australian conilurine rodents. Biol. Conserv..

[4] Beveridge I. (2020). Mammal parasites in arid Australia. Int. J. Parasitol. Parasites Wildl..

[5] Simons D., Attfield L.A., Jones K.E., Watson-Jones D., Kock R. (2023). Rodent trapping studies as an overlooked information source for understanding endemic and novel zoonotic spillover. PLoS Neglected Trop. Dis..

[6] Palmer B.J., Valentine L.E., Page M., Hobbs R.J. (2020). Translocations of digging mammals and their potential for ecosystem restoration: A review of goals and monitoring programmes. Mammal. Rev..

[7] Morris K., Page M., Kay R., Renwick J., Desmond A., Comer S., Burbidge A., Kuchling G., Sims C., Armstrong D.P., Hayward M.W., Moro D., Seddon P.J. (2015). Forty years of fauna translocations in Western Australia: Lessons learned. Advances in Reintroduction Biology of Australian and New Zealand Fauna.

[8] Han B.A., Schmidt J.P., Bowden S.E., Drake J.M. (2015). Rodent reservoirs of future zoonotic diseases. Proc. Natl. Acad. Sci. USA.

[9] Albery G.F., Becker D.J. (2021). Fast-lived Hosts and Zoonotic Risk. Trends Parasitol..

[10] Han B.A., Kramer A.M., Drake J.M. (2016). Global Patterns of Zoonotic Disease in Mammals. Trends Parasitol..

[11] Meerburg B.G., Singleton G.R., Kijlstra A. (2009). Rodent-borne diseases and their risks for public health. Crit. Rev. Microbiol..

[12] Albery G.F., Eskew E.A., Ross N., Olival K.J. (2020). Predicting the global mammalian viral sharing network using phylogeography. Nat. Commun..

[13] Brennan R.N., Paulson S.L., Escobar L.E. (2024). Estimating pathogen-spillover risk using host-ectoparasite interactions. Ecol. Evol..

[14] International Union for Conservation of Nature (2013). Guidelines for Reintroductions and Other Conservation Translocations. https://iucn-ctsg.org/project/new-rsg-re-introductions-guidelines-2013/.

[15] Morris K., Speldewinde P., Orell P. (2000). Djoongari (Shark Bay Mouse) Recovery Plan. Western Australian Wildlife Management Program No. 17. https://www.dcceew.gov.au/sites/default/files/documents/pseudomys-fieldi.pdf.

[16] Short J., Copley P., Ruykys L., Morris K., Read J., Moseby K. (2019). Review of translocations of the greater stick-nest rat (*Leporillus conditor*): Lessons learnt to facilitate ongoing recovery. Wildl. Res..

[17] Rayner K., Lohr C.A., Garretson S., Speldewinde P. (2021). Two species, one island: Retrospective analysis of threatened fauna translocations with divergent outcomes. PLoS ONE.

[18] Cowen S., Sims C., Ottewell K., Knox F., Friend T., Mills H., Garretson S., Rayner K., Gibson L. (2023). Return to 1616: Multispecies Fauna Reconstruction Requires Thinking Outside the Box. Animals.

[19] Short J., Richards J.D., O’Neill S. (2018). Reintroduction of the greater stick-nest rat (*Leporillus conditor*) to Heirisson Prong, Shark Bay: An unsuccessful attempt to establish a mainland population. Aust. Mammal..

[20] Grinde B. (2013). Herpesviruses: Latency and reactivation—Viral strategies and host response. J. Oral Microbiol..

[21] Ehlers B., Küchler J., Yasmum N., Dural G., Voigt S., Schmidt-Chanasit J., Jäkel T., Matuschka F.R., Richter D., Essbauer S. (2007). Identification of novel rodent herpesviruses, including the first gammaherpesvirus of *Mus musculus*. J. Virol..

[22] Knox F., Stephens N., Keatley S., Ash A., Douch J., Cowen S., Rayner K., Vaughan-Higgins R. (2025). Making the most of mortalities: Novel host-parasite records in a sandy inland mouse (*Pseudomys hermannsburgensis*). Int. J. Parasitol. Parasites Wildl..

[23] Campbell R.W., Carley J.G., Doherty R.L., Domrow R., Filippich C., Gorman B.M., Karabatsos N. (1977). Mossman virus, a paramyxovirus of rodents isolated in Queensland. Search.

[24] Jun M., Karabatsos N., Johnson R. (1977). A new mouse paramyxovirus (J virus). Aust. J. Exp. Biol. Med. Sci..

[25] Miller P.J., Boyle D.B., Eaton B.T., Wang L.-F. (2003). Full-length genome sequence of Mossman virus, a novel paramyxovirus isolated from rodents in Australia. Virology.

[26] Averis S., Thompson R., Lymbery A., Wayne A., Morris K., Smith A. (2009). The diversity, distribution and host-parasite associations of trypanosomes in Western Australian wildlife. Parasitology.

[27] Egan S.L., Taylor C.L., Austen J.M., Banks P.B., Ahlstrom L.A., Ryan U.M., Irwin P.J., Oskam C.L. (2020). Molecular identification of the *Trypanosoma* (*Herpetosoma*) *lewisi* clade in black rats (*Rattus rattus*) from Australia. Parasitol. Res..

[28] Wyatt K.B., Campos P.F., Gilbert M.T., Kolokotronis S.O., Hynes W.H., DeSalle R., Ball S.J., Daszak P., MacPhee R.D., Greenwood A.D. (2008). Historical mammal extinction on Christmas Island (Indian Ocean) correlates with introduced infectious disease. PLoS ONE.

[29] Austen J., Jefferies R., Friend J., Ryan U., Adams P., Reid S. (2009). Morphological and molecular characterization of *Trypanosoma copemani* n. sp. (*Trypanosomatidae*) isolated from Gilbert’s potoroo (*Potorous gilbertii*) and quokka (*Setonix brachyurus*). Parasitology.

[30] Smith A., Clark P., Averis S., Lymbery A., Wayne A., Morris K., Thompson R. (2008). Trypanosomes in a declining species of threatened Australian marsupial, the brush-tailed bettong *Bettongia penicillata* (Marsupialia: Potoroidae). Parasitology.

[31] Cassell G.H. (1982). The Pathogenic Potential of Mycoplasmas: *Mycoplasma pulmonis* as a Model. Rev. Infect. Dis..

[32] Mackie J.T., Booth R., Caton W., Stevenson R. (2001). Concurrent infection with cilia-associated respiratory bacillus and mycoplasmas in spinifex hopping-mice (*Notomys alexis*) with pneumonia. Aust. Vet. J..

[33] Smith A.L., Singleton G.R., Hansen G.M., Shellam G. (1993). A serologic survey for viruses and *Mycoplasma pulmonis* among wild house mice (*Mus domesticus*) in southeastern Australia. J. Wildl. Dis..

[34] Cowen S., Rayner K., Sims C. (2021). Dirk Hartog Island National Park Ecological Restoration Project: Stage Two–Year Three.

[35] Cowen S., Rayner K., Sims C., Van der Weyde L. (2022). Dirk Hartog Island National Park Ecological Restoration Project: Stage Two–Year Four.

[36] Christopher M.M. (2004). Evaluation of the Blood Smear.

[37] Brooks M.B., Harr K.E., Seelig D.M., Wardrop K.J., Weiss D.J. (2022). Schalm’s Veterinary Hematology, 7th Edition.

[38] Dunnet G.M., Nardon D. (1974). A monograph of Australian fleas (*Siphonaptera*). Aust. J. Zool. Suppl. Ser..

[39] Roberts F. (1963). A systematic study of the Australian species of the genus *Haemaphysalis* Koch (Acarina: Ixodidae). Aust. J. Zool..

[40] Roberts F.H.S. (1970). Australian Ticks.

[41] Halliday B. (2024). A collection of mites in the family Laelapidae from rodents in Western Australia. Rec. West. Aust. Mus..

[42] Domrow R. (1987). Acari Mesostigmata parasitic on Australian vertebrates: An annotated checklist, keys and bibliography. Invertebr. Syst..

[43] Kim K.C., Ludwig H.W. (1978). The family classification of the Anoplura. Sys. Entomol..

[44] Maslov D.A., Lukeš J., Jirku M., Simpson L. (1996). Phylogeny of trypanosomes as inferred from the small and large subunit rRNAs: Implications for the evolution of parasitism in the trypanosomatid protozoa. Mol. Biochem. Parasitol..

[45] Beard A.W., Maggi R.G., Kennedy-Stoskopf S., Cherry N.A., Sandfoss M.R., DePerno C.S., Breitschwerdt E.B. (2011). *Bartonella* spp. in feral pigs, southeastern United States. Emerg. Infect. Dis..

[46] VanDevanter D.R., Warrener P., Bennett L., Schultz E.R., Coulter S., Garber R.L., Rose T.M. (1996). Detection and analysis of diverse herpesviral species by consensus primer PCR. J. Clin. Microbiol..

[47] Tong S., Chern S.W., Li Y., Pallansch M.A., Anderson L.J. (2008). Sensitive and broadly reactive reverse transcription-PCR assays to detect novel paramyxoviruses. J. Clin. Microbiol..

[48] van Kuppeveld F.J., van der Logt J.T., Angulo A.F., van Zoest M.J., Quint W.G., Niesters H.G., Galama J.M., Melchers W.J. (1992). Genus- and species-specific identification of mycoplasmas by 16S rRNA amplification. Appl. Environ. Microbiol..

[49] Kumar S., Stecher G., Suleski M., Sanderford M., Sharma S., Tamura K. (2024). MEGA12: Molecular Evolutionary Genetic Analysis version 12 for adaptive and green computing. Mol. Biol. Evol..

[50] R Core Team (2024). R: A Language and Environment for Statistical Computing. R Foundation for Statistical Computing. https://www.R-project.org/.

[51] Ullman-Culleré M.H., Foltz C.J. (1999). Body condition scoring: A rapid and accurate method for assessing health status in mice. Comp. Med..

[52] Tulk M.L., Stannard H.J., Old J.M. (2016). Haematology and serum biochemistry in captive Australian native murids: Black-footed tree rat (*Mesembriomys gouldii*) and greater stick-nest rat (*Leporillus conditor*). SpringerPlus.

[53] Wang W., Durden L.A., Weaver H., Shao R. (2021). Eight new species of sucking lice (Psocodea: Phthiraptera) from endemic murine rodents in Australia and an updated identification key. J. Med. Entomol..

[54] Votýpka J., Stříbrná E., Modrý D., Bryja J., Bryjová A., Lukeš J. (2022). Unexpectedly high diversity of trypanosomes in small sub-Saharan mammals. Int. J. Parasitol..

[55] Kimura M. (1980). A simple method for estimating evolutionary rates of base substitutions through comparative studies of nucleotide sequences. J. Mol. Evol..

[56] Knowles S.C.L., Fenton A., Pedersen A.B. (2012). Epidemiology and fitness effects of wood mouse herpesvirus in a natural host population. J. Gen. Virol..

[57] Dickens M.J., Delehanty D.J., Michael Romero L. (2010). Stress: An inevitable component of animal translocation. Biol. Conserv..

[58] Blumstein D.M., MacManes M.D. (2023). When the tap runs dry: The physiological effects of acute experimental dehydration in *Peromyscus eremicus*. J. Exp. Biol..

[59] Chudnovskiy A., Mortha A., Kana V., Kennard A., Ramirez J.D., Rahman A., Remark R., Mogno I., Ng R., Gnjatic S. (2016). Host-Protozoan Interactions Protect from Mucosal Infections through Activation of the Inflammasome. Cell.

[60] Hunter-Barnett S., Viney M. (2024). Gut protozoa of wild rodents—A meta-analysis. Parasitology.

[61] Stringer A.P., Linklater W. (2014). Everything in Moderation: Principles of Parasite Control for Wildlife Conservation. BioScience.

[62] Carlson C.J., Hopkins S., Bell K.C., Doña J., Godfrey S.S., Kwak M.L., Lafferty K.D., Moir M.L., Speer K.A., Strona G. (2020). A global parasite conservation plan. Biol. Conserv..

[63] Whinfield J., Warren K., Vogelnest L., Vaughan-Higgins R. (2024). Applying a modified streamlined disease risk analysis framework to a platypus conservation translocation, with special consideration for the conservation of ecto- and endoparasites. Int. J. Parasitol. Parasites Wildl..

[64] Kwak M.L., Burns P.A., Hastriter M.W., Jenkins Shaw J., Nakao R. (2024). The threatened Pookila (*Pseudomys novaehollandiae*) hosts a diverse macrobiome of arthropods at varying risks of co-extinction. J. Insect Conserv..

[65] Ho J., Changbunjong T., Weluwanarak T., Hussain S., Sparagano O. (2021). The pests of a pest: A systematic review of ectoparasitic fauna among synanthropic rodents in the 21st century with meta-analysis. Acta Trop..

[66] Ortiz P.A., Garcia H.A., Lima L., da Silva F.M., Campaner M., Pereira C.L., Jittapalapong S., Neves L., Desquesnes M., Camargo E.P. (2018). Diagnosis and genetic analysis of the worldwide distributed *Rattus*-borne *Trypanosoma* (*Herpetosoma*) *lewisi* and its allied species in blood and fleas of rodents. Infect. Genet. Evol..

[67] Desquesnes M., Gonzatti M., Sazmand A., Thévenon S., Bossard G., Boulangé A., Gimonneau G., Truc P., Herder S., Ravel S. (2022). A review on the diagnosis of animal trypanosomoses. Parasites Vectors.

[68] Papagni R., Novara R., Minardi M.L., Frallonardo L., Panico G.G., Pallara E., Cotugno S., Ascoli Bartoli T., Guido G., De Vita E. (2023). Human African Trypanosomiasis (sleeping sickness): Current knowledge and future challenges. Front. Trop. Dis..

[69] Coura J.R., Borges-Pereira J. (2010). Chagas disease: 100 years after its discovery. A systemic review. Acta Trop..

[70] Truc P., Büscher P., Cuny G., Gonzatti M.I., Jannin J., Joshi P., Juyal P., Lun Z.-R., Mattioli R., Pays E. (2013). Atypical Human Infections by Animal Trypanosomes. PLoS Neglected Trop. Dis..

[71] Shaw G.L., Dusanic D.G. (1973). *Trypanosoma lewisi*: Termination of pregnancy in the infected rat. Exp. Parasitol..

[72] Budovsky A., Prinsloo I., El-On J. (2006). Pathological developments mediated by cyclophosphamide in rats infected with *Trypanosoma lewisi*. Parasitol. Int..

[73] Babyesiza W.S., Katakweba A., Fornuskova A., Ssuunaf J., Akoth S., Mpagi J., Gouy de Bellocq J., Bryja J., Votypka J. (2023). Trypanosome diversity in small mammals in Uganda and the spread of *Trypanosoma lewisi* to native species. Parasitol. Res..

[74] Gundi V.A., Taylor C., Raoult D., La Scola B. (2009). *Bartonella rattaustraliani* sp. nov.; *Bartonella queenslandensis* sp. nov. and *Bartonella coopersplainsensis* sp. nov.; identified in Australian rats. Int. J. Syst. Evol. Microbiol..

[75] Egan S.L., Taylor C.L., Banks P.B., Northover A.S., Ahlstrom L.A., Ryan U.M., Irwin P.J., Oskam C.L. (2021). The bacterial biome of ticks and their wildlife hosts at the urban– wildland interface. Microb. Genom..

[76] Dybing N.A., Jacobson C., Irwin P., Algar D., Adams P.J. (2016). *Bartonella* Species Identified in Rodent and Feline Hosts from Island and Mainland Western Australia. Vector-Borne Zoonotic Dis..

[77] Jacomo V., Kelly P.J., Raoult D. (2002). Natural history of *Bartonella* infections (an exception to Koch’s postulate). Clin. Diagn. Lab. Immunol..

[78] Deng H., Le Rhun D., Buffet J.-P.R., Cotté V., Read A., Birtles R.J., Vayssier-Taussat M. (2012). Strategies of exploitation of mammalian reservoirs by *Bartonella* species. Vet. Res..

[79] Wildlife Health Australia (2023). *Bartonella* and Australian Wildlife. Wildlife Health Australia. https://wildlifehealthaustralia.com.au/Portals/0/ResourceCentre/FactSheets/PublicHealth/Bartonella_in_Australian_wildlife.pdf.

[80] Krügel M., Król N., Kempf V.A.J., Pfeffer M., Obiegala A. (2022). Emerging rodent-associated Bartonella: A threat for human health?. Parasites Vectors.

[81] Kaminskienė E., Paulauskas A., Balčiauskas L., Radzijevskaja J. (2022). *Bartonella* spp. detection in laelapid (*Mesostigmata*: *Laelapidae*) mites collected from small rodents in Lithuania. J. Vector Ecol..

[82] Loftis A.D., Reeves W.K., Szumlas D.E., Abbassy M.M., Helmy I.M., Moriarity J.R., Dasch G.A. (2006). Surveillance of Egyptian fleas for agents of public health significance: *Anaplasma*, *Bartonella*, *Coxiella*, *Ehrlichia*, *Rickettsia*, and *Yersinia pestis*. Am. J. Trop. Med. Hyg..

[83] Zhou S., Nian Y., Zeng Z., Han T., Liu W., Zheng K., Xiao F. (2024). Epidemiological survey and genetic diversity of *Bartonella* in fleas collected from rodents in Fujian Province, Southeast China. Parasites Vectors.

[84] Moody A.H., Chiodini P.L. (2000). Methods for the detection of blood parasites. Clin. Lab. Haematol..

[85] Azab W., Dayaram A., Greenwood A.D., Osterrieder N. (2018). How Host Specific Are Herpesviruses? Lessons from Herpesviruses Infecting Wild and Endangered Mammals. Annu. Rev. Virol..

[86] Ntumvi N.F., Mbala Kingebeni P., Tamoufe U., Kumakamba C., Ndze V., Ngay Lukusa I., LeBreton M., Atibu Losoma J., Le Doux Diffo J., N’Kawa F. (2018). High Herpesvirus Diversity in Wild Rodent and Shrew Species in Central Africa. Intervirology.

[87] Fisher D.O. (2011). Cost, effort and outcome of mammal rediscovery: Neglect of small species. Biol. Conserv..

[88] Estep R.D., Wong S.W. (2013). Rhesus macaque rhadinovirus-associated disease. Curr. Opin. Virol..

[89] François S., Vidick S., Sarlet M., Michaux J., Koteja P., Desmecht D., Stevenson P.G., Vanderplasschen A., Gillet L. (2010). Comparative study of murid gammaherpesvirus 4 infection in mice and in a natural host, bank voles. J. Gen. Virol..

[90] Cesarman E., Damania B., Krown S.E., Martin J., Bower M., Whitby D. (2019). Kaposi sarcoma. Nat. Rev. Dis. Primers.

[91] Nash A.A., Dutia B.M., Stewart J.P., Davison A.J. (2001). Natural history of murine gammaherpesvirus infection. Philos. Trans. R. Soc. London. Ser. B Biol. Sci..

[92] Deim Z., Szeredi L., Tompó V., Egyed L. (2006). Detection of bovine herpesvirus 4 in aborted bovine placentas. Microb. Pathog..

[93] Doğan F., Farzani T.A., Alkan F., Dağalp S.B., Babaoglu A.R. (2020). An assessment of bovine herpes virus 4 as a causative agent in abortions and neonatal death. Onderstepoort J. Vet. Res..

[94] Williams-Kelly K.S., Berry L., Branch K., Cowen S., Garretson S., Holland G.J., Ladd R., Olds L., Rayner K., Sims C. (2023). Physiological response after translocation differs between source populations in a threatened mammal. R. Soc. Open Sci..

[95] Wildlife Health Australia (2018). National Wildlife Biosecurity Guidelines. Wildlife Health Australia.

[96] Singleton G.R., Smith A.L., Shellam G., Fitzgerald N., Müller W.J. (1993). Prevalence of viral antibodies and helminths in field populations of house mice (*Mus domesticus*) in southeastern Australia. Epidemiol. Infect..

[97] Moro D., Lawson M.A., Hobbs R.P., Thompson R.C.A. (2003). Pathogens of house mice on arid Boullanger Island and subantarctic Macquarie Island, Australia. J. Wildl. Dis..

[98] Thomas L. (1967). The neurotoxins of *M. neurolyticum* and *M. Gallisepticum*. Ann. N. Y. Acad. Sci..

[99] Langan G.P., Lohmiller J.J., Swing S.P., Wardrip C.L. (2000). Respiratory diseases of rodents and rabbits. Vet. Clin. N. Am. Small Anim. Pract..

[100] Kirchhoff H., Binder A., Runge M., Meier B., Jacobs R., Busche K. (1989). Pathogenetic mechanisms in the *Mycoplasma arthritidis* polyarthritis of rats. Rheumatol. Int..

[101] Naot Y., Sharf M., Klein A. (1978). *Mycoplasma* Infection in Pregnant Rats: Low Viability of Foetuses and Newborn OffSpring. J. Med. Microbiol..

[102] Rothenburger J., Himsworth C., Clifford C., Ellis J., Treuting P., Leighton F. (2015). Respiratory pathology and pathogens in wild urban rats (*Rattus norvegicus* and *Rattus rattus*). Vet. Pathol..

[103] Grange Z.L., Goldstein T., Johnson C.K., Anthony S., Gilardi K., Daszak P., Olival K.J., O’Rourke T., Murray S., Olson S.H. (2021). Ranking the risk of animal-to-human spillover for newly discovered viruses. Proc. Natl. Acad. Sci. USA.

[104] Larsen B.B., Gryseels S., Otto H.W., Worobey M. (2022). Evolution and Diversity of Bat and Rodent Paramyxoviruses from North America. J. Virol..

[105] Thibault P.A., Watkinson R.E., Moreira-Soto A., Drexler J.F., Lee B. (2017). Zoonotic Potential of Emerging Paramyxoviruses: Knowns and Unknowns. Adv. Virus Res..

[106] Ghawar W., Pascalis H., Bettaieb J., Mélade J., Gharbi A., Snoussi M.A., Laouini D., Goodman S.M., Ben Salah A., Dellagi K. (2017). Insight into the global evolution of Rodentia associated *Morbilli-related* paramyxoviruses. Sci. Rep..

[107] Copley P. (1999). Review of the Recovery Plan for Greater Stick-Nest Rat, Leporillus conditor.

[108] Robertson H. Wildlife conservation and Perth zoo. Proceedings of the National Wildlife Rehabilitation Conference.

[109] Kovacs K.A., Lam Y.M., Pater J.L. (2001). Bilateral massive adrenal hemorrhage. Assessment of putative risk factors by the case-control method. Medicine.

[110] Feldt-Rasmussen U. (2024). Extensive Expertise in Endocrinology: Adrenal crisis in assisted reproduction and pregnancy. Eur. J. Endocrinol..

[111] Hofer M.A. (1970). Cardiac and respiratory function during sudden prolonged immobility in wild rodents. Psychosom. Med..

[112] Liu J., Hakucho A., Liu X., Fujimiya T. (2016). Acute restraint stress provokes sudden cardiac death in normotensive rats and enhances susceptibility to arrhythmogenic effects of adrenaline in spontaneously hypertensive rats. Leg. Med..

