# Supplementary material for: Giving a Rat’s: Acquisition of Baseline Health Data on Native Australian Rodents During a Conservation Translocation
Source: Animals (Basel). 2026 Sep 27;16(19):3044. doi: 10.3390/ani16193044 (PMC13663421; doi:10.3390/ani16193044)
Supplement: Supplementary file 1 [file animals-16-03044-s001.zip › animals-4499751-supplementary.pdf]

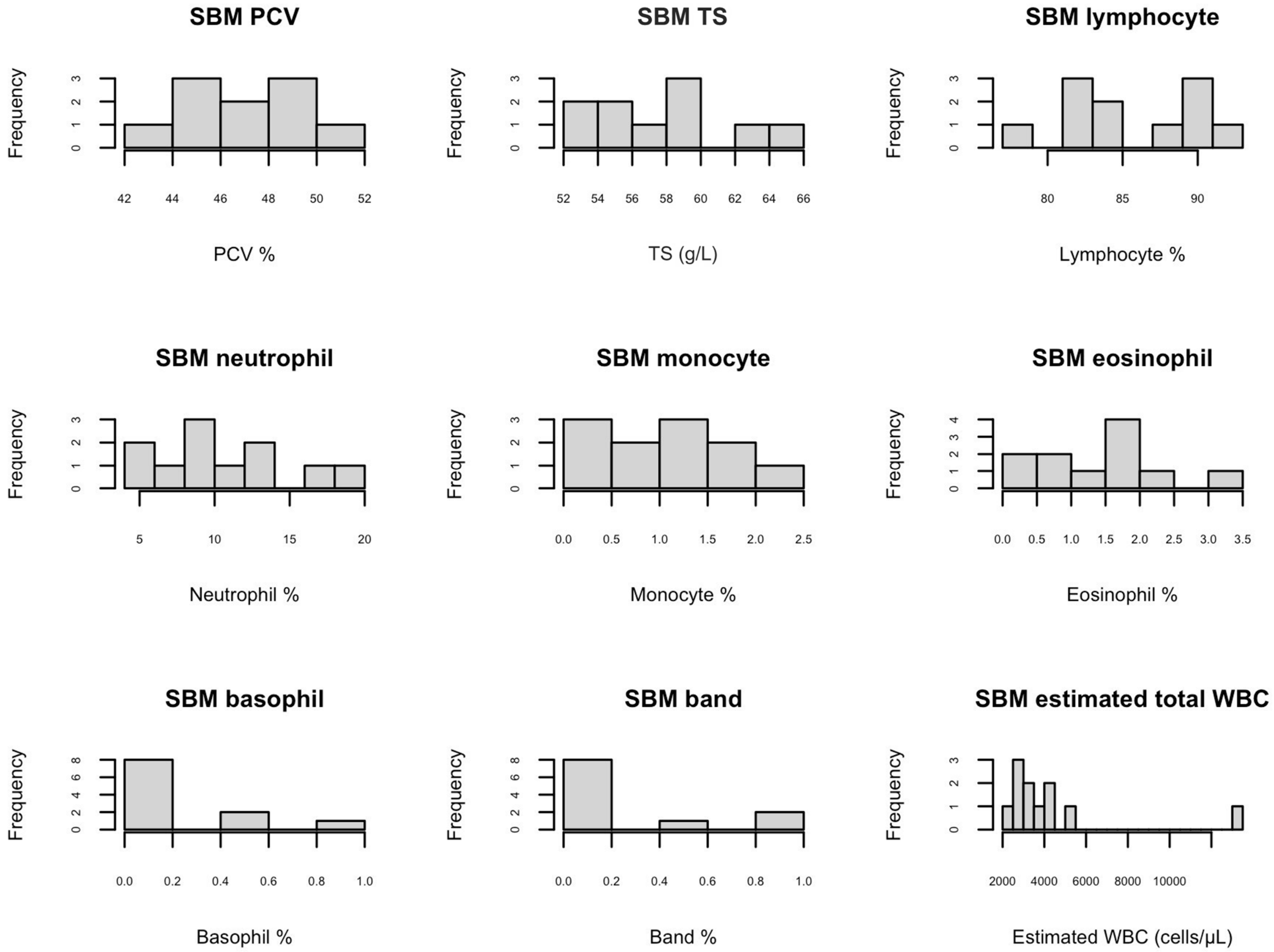

Figure S1: Histograms of haematology results of Shark Bay mice. TS = total solids; PCV = packed cell volume; WBC = white blood cell.

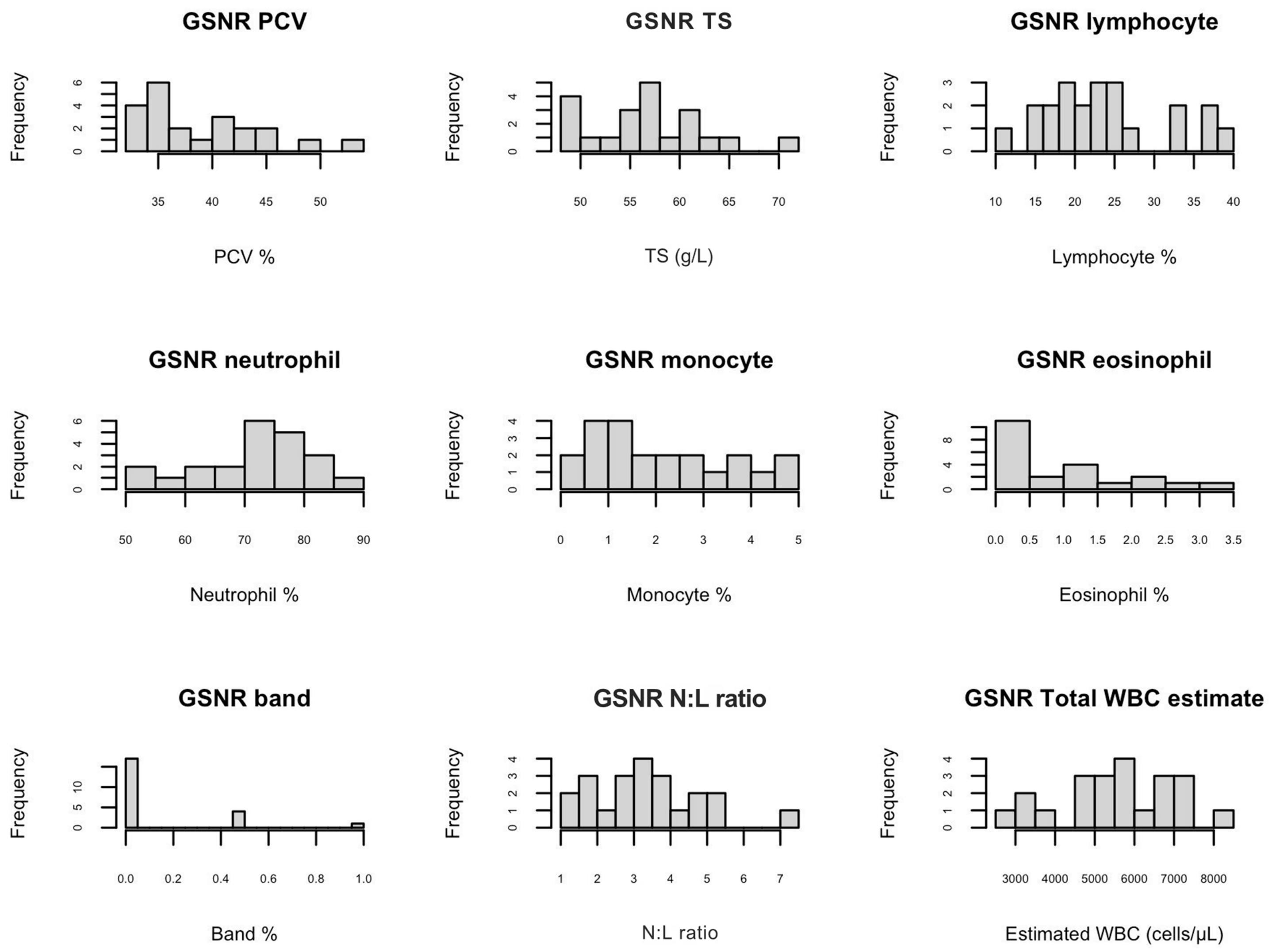

Figure S2: Histograms of haematology results of greater stick-nest rats. TS = total solids; PCV = packed cell volume; WBC = white blood cell.

Table S1: Faecal parasitology apparent prevalence (AP) across species and cohorts**.** 37.2% of GSNR and 27% of SBM were positive for at least one taxon by faecal flotation. There was no statistically significant difference of apparent prevalence of faecal parasites between cohorts of GSNR (Fisher's exact, $p = 0.158$), or SBM ($X^2 = 0.12$, df = 1, $p = 0.73$), noting only a small number of faecal samples ($n = 12$) were collected from the Franklin Islands. Species identification was not possible through our methods, although in most cases, parasites were identified to a family/genus level.

**Taxa**	**GSNR** AP % (95% CI and total)	**Salutation Island** AP % (95% CI and total)	**Franklin Islands** AP % (95% CI and total)	**SBM** AP % (95% CI and total)	**Bernier Island** AP % (95% CI and total)	**Northwest Island** AP % (95% CI and total)
**Any**	37.21 (24.38–52.14; *n* = 16/43)	45.16 (29.16-62.23; *n* = 14/31)	16.67 (4.7-44.8; *n* = 2/12)	26.97 (18.84-37; *n* = 24/89)	24.49 (14.6-38.09; *n* = 12/49)	30 (18.07-45.43; *n* = 12/40)
Entamoeba	27.91 (16.75-42.69; *n* = 12/43)	35.48 (21.12-53.05; *n* = 11/31)	8.33 (0.43-35.39; *n* = 1/12)	2.25 (0.62-7.83; *n* = 2/89)	2.04 (0.1-10.69; *n* = 1/49)	2.5 (0.13-12.88; *n* = 1/40)
Unidentified protozoan	20.93 (11.42-35.21; *n* = 9/43)	25.81 (13.7-43.25; *n* = 8/31)	8.33 (0.43-35.39; *n* = 1/12)	1.12 (0.06-6.09; *n* = 1/89)	2.04 (0.1-10.69; *n* = 1/49)	0 (0-8.76; *n* = 0/40)
Coccidian	2.33 (0.12-12.06; *n* = 1/43)	3.22 (0.17-16.19; *n* = 1/31)	0 (0-24.25; *n* = 0/12)	-	-	-
Chilomastix	6.98 (2.4-18.61; *n* = 3/43)	9.68 (3.35-24.9; *n* = 3/31)	0 (0-24.25; *n* = 0/12)	-	-	-
Trichomonidae	-	-	-	21.35 (14.11-30.95, *n* = 19/89)	18.37 (9.98-31.36; *n* = 9/49)	25 (14.19-4019; *n* = 10/40)
Spiruroid	-	-	-	1.12 (0.06-6.09; *n* = 1/89)	0 (0-7.27; *n* = 0/49)	2.5 (0.13-12.88; *n* = 1/40)
Protospirura or *Mastophurus*	-	-	-	1.12 (0.06-6.09; *n* = 1/89)	0 (0-7.27; *n* = 0/49)	2.5 (0.13-12.88; *n* = 1/40)
Unidentified cestode	-	-	-	1.12 (0.06-6.09; *n* = 1/89)	2.04 (0.1-10.69; *n* = 1/49)	0 (0-8.76; *n* = 0/40)
Multiple burdens	16.28 (8.12-29.97; *n* = 7/43)	22.58 (11.4-39.81; *n* = 7/31)	0 (0-24.25; *n* = 0/12)	2.25 (0.62-7.83; *n* = 2/89)	2.04 (0.1-10.69; *n* = 1/49)	2.5 (0.13-12.88; *n* = 1/40)

Table S2: Nucleotide sequences obtained from amplification of the *Orthoherpesviridae* DNA polymerase catalytic subunit gene region from rodent swabs collected during this study.

Species	Nucleotide sequence
SBM	ATGGCCGGCGGTCGCGAGCGACATATCCCTGCTGATCTTGGTGATAAAAGCGGC
GSNR	ACGGGGGTGGCCAGTGGCATCTTGCCCTGCGTCAAGATCGCCGAGACGGTGACTTTCGTGGGCCGACGGATGCTGGGAG<br>CCTCGATGGACCTGGTGAATTCCCTGTCGCTGGAGCGACTCGAGGAGATTCACGGGGCGCCGCTGCCGCGCGGCGAGGG<br>GGCCTCGCTCCGAGTGATCTACGGCGACACGGACTCTG

Table S3: Nucleotide sequences obtained from amplification of the *Mollicutes* 16S rRNA gene region from rodent swabs collected during this study.

Species	Year sourced	Nucleotide sequence
SBM	2021/2022	GGTAGTCCACGCCGTAAACGATGATCATTAGTTGGTAGAAATAATTTACTAACGCAGCTAACGCATTAAATGATCCGCCT GAGTAGTATGCTCGCAAGAGTGAAACTTAAAGGAATTGACGGGGACCCGCACAAGCGGTGGAGCATGTGGTTTAATTTGA AGATACGCGTAGAACCTTACCCACTCTTGACATCCTTTGCAAAGTTATAGAGATATAATG
GSNR	2021	GCCCCCCGTGAATAATATGCTCACGGGAGTGACTCTTAGGAGATTTGACGCAGACCCACACACGGGGTGGAACGTGTTTT TTATTTTGAATATACGCATAAAATCTTACCCACTCTTGACCTCTTCTGCAACTATATAAATATATAGTG
GSNR	2021/2022	GGTAGTCCACGCCGTAAACGATGATCATTAGTTGGTAGAAGAATTTACCGACGCAGCTAACGCATTAAATGATCCGCCTG AGTAGTATGCTCGCAAGAGTGAAACTTAAAGGAATTGACGGGGACCCGCACAAGCGGTGGAGCATGTGGTTTAATTTGAA GATACGCGTAGAACCTTACCCACTCTTGACATCTTCTGCAAAAATATAGAGATATATTG
GSNR	2022	ATTCATCGGCGCAGCTAACGCATTAAATGATCCGCCTGAGTAGTACGTTCGCAAGAATAAAACTTAAAGGAATTGACGGGGAT CCGCACAAGCGGTGGAGCATGTGGTTTAATTTGAAGATACGCGTAGAACCTTACCCACTCTTGACTTCTTCTGCAATGCTATGG AGACATAGCG
